# Gene Amplification of *CYP51B*: a New Mechanism of Resistance to Azole Compounds in Trichophyton indotineae

**DOI:** 10.1128/aac.00059-22

**Published:** 2022-05-12

**Authors:** Tsuyoshi Yamada, Takashi Yaguchi, Mari Maeda, Mohamed Mahdi Alshahni, Karine Salamin, Emmanuella Guenova, Marc Feuermann, Michel Monod

**Affiliations:** a Teikyo University Institute of Medical Mycology, Tokyo, Japan; b Asia International Institute of Infectious Disease Control, Teikyo University, Tokyo, Japan; c Medical Mycology Research Center, Chiba University, Chiba, Japan; d Department of Dermatology, Centre Hospitalier Universitaire Vaudois, Lausanne, Switzerland; e Faculty of Biology and Medicine, University of Lausanne, Lausanne, Switzerland; f Swiss-Prot Group, SIB Swiss Institute of Bioinformatics, Geneva, Switzerland

**Keywords:** dermatophytes, *Trichophyton mentagrophytes* type VIII, *Trichophyton indotineae*, itraconazole, voriconazole, resistance, ABC transporters, *CYP51B*

## Abstract

Trichophyton indotineae causes dermatophytosis that is resistant to terbinafine and azole compounds. The aim of this study was to determine the mechanisms of resistance to itraconazole (ITC) and voriconazole (VRC) in strains of *T. indotineae*. Two azole-sensitive strains (ITC MIC < 0.125 μg/mL; VRC MIC < 0.06 μg/mL) and four azole-resistant strains (ITC MIC ≥ 0.5 μg/mL; VRC MIC ≥ 0.5 μg/mL) were used for the investigation. The expression of *MDR* genes encoding multidrug transporters of the ABC family for which orthologs have been identified in Trichophyton rubrum and those of *CYP51A* and *CYP51B* encoding the targets of azole antifungal compounds were compared between susceptible and resistant strains. *TinMDR3* and *TinCYP51B* were overexpressed in *T. indotineae* resistant strains. Only small differences in susceptibility were observed between *TinMDR3* disruptants and parental strains overexpressing *TinMDR3.* Whole-genome sequencing of resistant strains revealed the creation of a variable number of *TinCYP51B* tandem repeats at the specific position of their genomes in three resistant strains. Downregulation of *TinCYP51B* by RNA interference (RNAi) restored the susceptibility of azole-resistant strains. In contrast, overexpression of *TinCYP51B* cDNA conferred resistance to a susceptible strain of *T. indotineae*. In conclusion, the reduced sensitivity of *T. indotineae* strains to azoles is mainly due to the overexpression of *TinCYP51B* resulting from additional copies of this gene.

## INTRODUCTION

The acquired resistance of dermatophytes to commonly used antifungal compounds is a serious emerging problem in several countries. In all recorded cases of Trichophyton rubrum, Trichophyton interdigitale, Trichophyton tonsurans, and Trichophyton indotineae (formally called T. interdigitale or Trichophyton mentagrophytes type VIII), resistance toward terbinafine was generated by amino acid substitutions in the squalene epoxidase (SQLE), a target molecule of terbinafine (1–5). These mutations are most often found at Leu393 and Phe397 in SQLE. In contrast, the resistance of one T. rubrum clinical strain to itraconazole (ITC) and voriconazole (VRC) was found to be associated with the overexpression of two genes (*TruMDR2* and *TruMDR3*) encoding multidrug transporters of the ABC family (6). Two other ABC transporters (TruMDR1 and TruMDR5) and two major facilitator superfamily transporters (TruMFS1 and TruMFS2) that are capable of operating as azole efflux pumps were found in T. rubrum, but these four transporters did not appear to be involved in the resistance of this strain.

While the resistance of T. rubrum to azoles remains exceptional, it seems to be common with *T. indotineae* isolated from skin dermatophytosis lesions in India (7, 8). Recently, 297 strains of *T. indotineae* were tested for susceptibility to ITC and VRC, and also for resistance to terbinafine (7). The MIC of ITC was reported to be around 0.016 μg/mL in 90% of the tested strains, while 10% of the strains showed reduced susceptibility to ITC, with a MIC of ≥0.5 μg/mL. The strains with reduced susceptibility to ITC were also less susceptible to VRC, and an abnormal distribution of MICs was observed for VRC, leading to the assumption of resistance mechanisms for strains with a VRC MIC of >0.25 μg/mL. Such resistance was not drug specific but was mediated by a shared mechanism of resistance (7).

Azole resistance in human-pathogenic fungi was first studied in Candida albicans and then in other yeasts and filamentous fungi, in particular, Aspergillus fumigatus. Several mechanisms leading to resistance have been described, including point mutations in genes encoding drug targets (9–12), overexpression of those targets (13, 14), and overexpression of genes encoding multidrug transporters involved in drug efflux (15–17). A combination of these three mechanisms results in an additive effect (18, 19). However, to date, there are no data on the possible azole resistance mechanisms in *T. indotineae* isolates. The aim of the present study was to elucidate the mechanisms of azole resistance to ITC and VRC in strains of *T. indotineae*. We found that resistance was due to overexpression of the *TinCYP51B* gene encoding sterol 14α-demethylase in three of the four low-azole-susceptible strains studied, which resulted from additional copies of this gene in tandem.

## RESULTS

**FIG 1:**
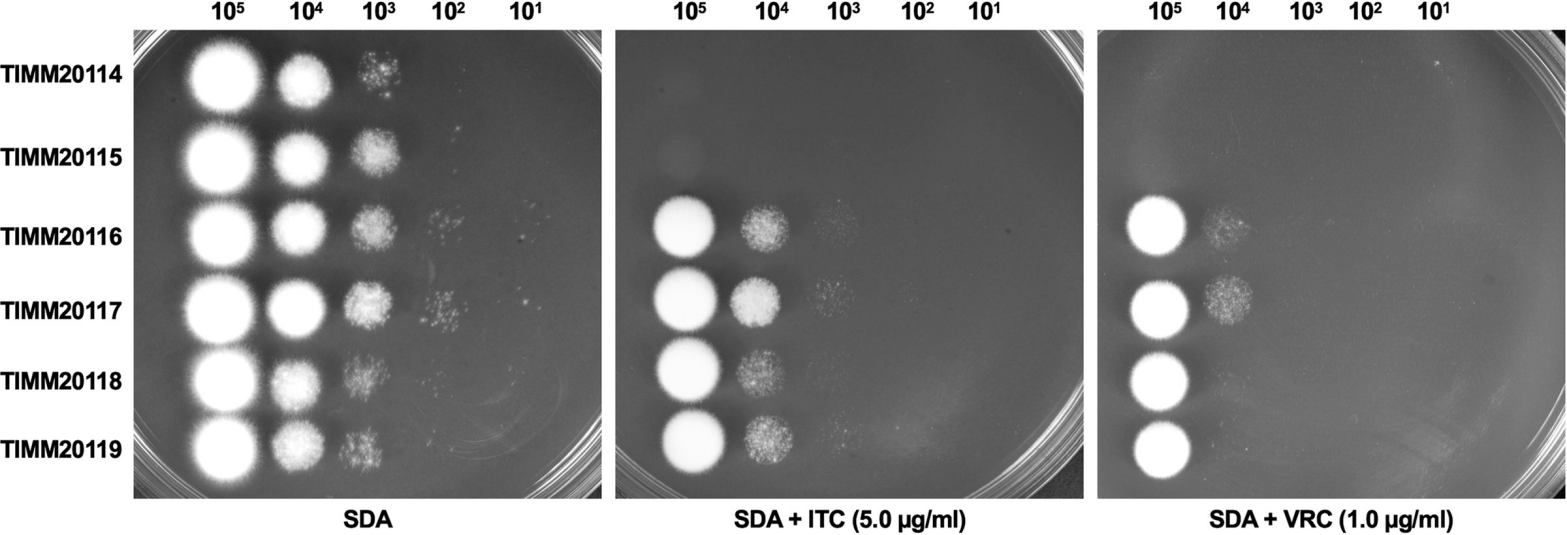
Evaluation of ITC and VRC susceptibility in six *T. indotineae* isolates by serial dilution drug susceptibility assays. *T. indotineae* spores were spotted at different dilutions on SDA plates, as described in Materials and Methods. The plates were incubated at 28°C for 3 to 5 days.

To study the mechanisms involved in resistance acquisition in *T. indotineae*, we focused on two strains that were deemed to be susceptible to ITC (ITC MIC < 0.125 μg/mL; VRC MIC < 0.06 μg/mL), called TIMM20114 and TIMM20115, and four strains that were deemed to have low susceptibility to ITC (ITC MIC ≥ 0.5 μg/mL; VRC MIC ≥ 0.5 μg/mL), called TIMM20116 to TIMM20119 (Table 1, Fig. 1).

**TABLE 1:** Phenotypic and genotypic characteristics of *T. indotineae* isolates used in this study

*T. indotineae* sp. and isolate no.^*a*^	Amino acid substitution in SQLE	ITC MIC_80_ (μg/mL)	VRC MIC_80_ (μg/mL)	Fold expression of *TinCYP51B* (mean ± SD)^*b*^	*TinCYP51B* copy no. within genome
TIMM20114 (**UKJ1676/17;** IFM 67092)	Ala448Thr	0.06	0.015	1	1
TIMM20115 (**UKJ1700/17;** IFM 67093)	Phe397Leu	0.06	0.03	1.1 ± 0.4	1
TIMM20116 (**UKJ1708/17;** IFM 67094)	Ala448Thr	1.0	1.0	34.0 ± 5.3	5
TIMM20117 (**200087/18;** IFM 67095)	Ala448Thr	0.5	0.5	9.6 ± 0.7	1
TIMM20118 (**UKJ1687/17;** IFM 67096)	Phe397Leu	0.5	1.0	35.0 ± 12.1	7
TIMM20119 (**200123/18;** IFM 67097)	Phe397Leu	1.0	1.0	68.5 ± 25.3	5

a All strains were from a previously published resistance study in India, with the numbering in bold (7). They were then preserved in the culture collection of Teikyo University Institute of Medical Mycology (TIMM) and Medical Mycology Research Center, Chiba University (IFM), through the National Bio-Resource Project, Japan (http://www.nbrp.jp/).

b Results represent expression levels from three independent real-time PCR experiments. Expression levels of *TinCYP51B* genes were indicated as relative fold changes compared to the CT mean of the data from TIMM20114.

### Point mutations in *TinCYP51B* without an effect on azole resistance.

**FIG 2:**
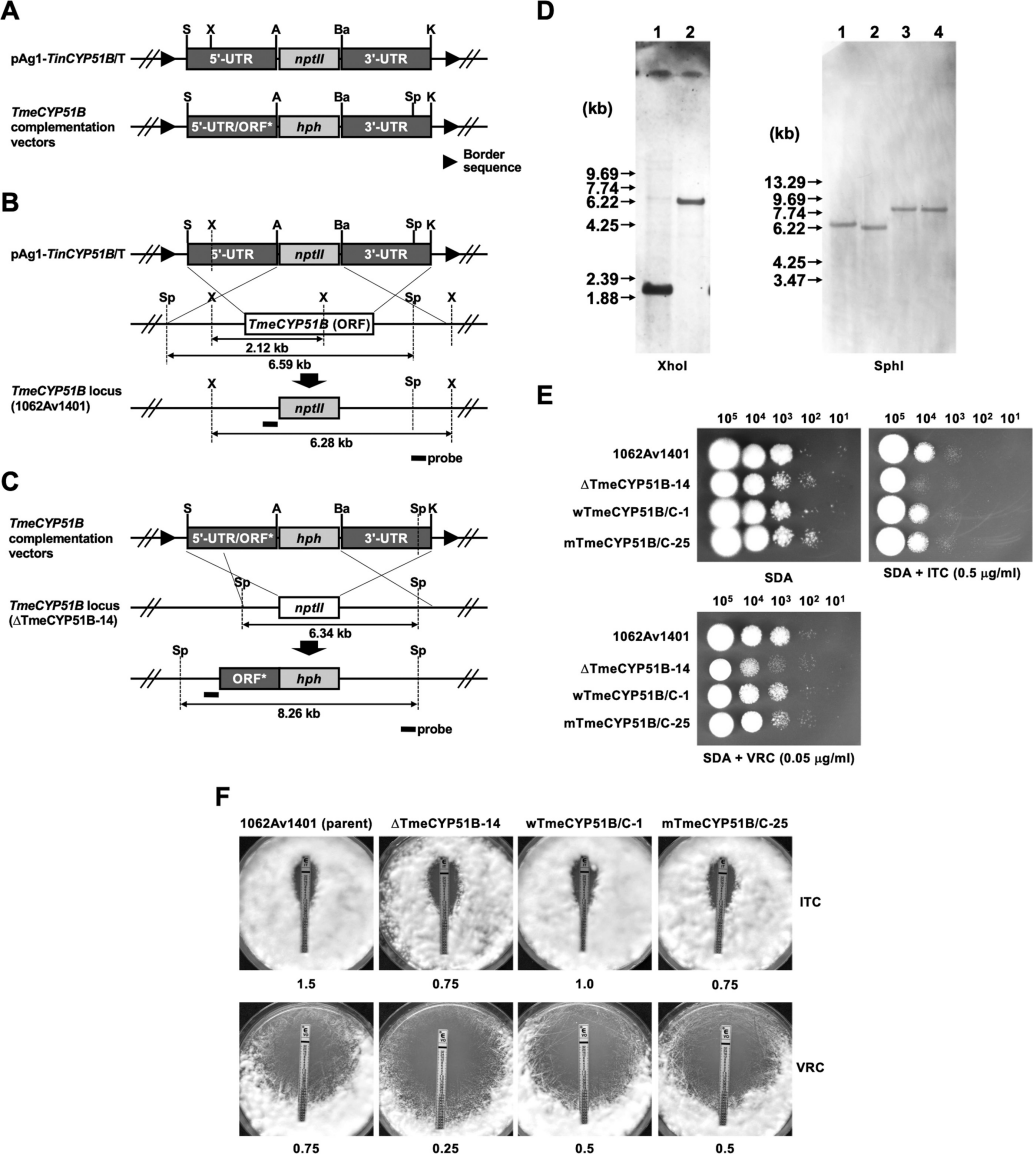
Disruption of the *CYP51B* homolog (*TmeCYP51B*) of T. mentagrophytes 1062Av1401 and subsequent reintroduction of the wild-type and mutated copy of *TmeCYP51B* by a gene replacement strategy. (A) Schematic representation of a binary *TinCYP51B*-targeting vector, pAg1-*TinCYP51B*/T. The *nptII* cassette is composed of Aspergillus nidulans *trpC* promoter (*PtrpC*), E. coli neomycin phosphotransferase gene (*nptII*), and A. fumigatus *cgrA* terminator (*TcgrA*). Border sequences are the specific regions that delineate the DNA to be transferred during Agrobacterium tumefaciens-mediated transformation. Restriction enzyme site: A, ApaI; Ba, BamHI; E, EcoRI; K, KpnI; P, PstI; S, SpeI; X, XhoI. (B) Schematic representation of the *TmeCYP51B* locus before and after homologous recombination. (C) Schematic representation of the *TmeCYP51B* locus in the ΔTmeCYP51B-14 before and after complementation by two kinds of binary vectors pAg1-*wTmeCYP51B*/C and pAg1-*mTmeCYP51B*/C. DNA fragments (TmeCYP51B1 and TmeCYP51B2) containing the 5′ UTR of the *TmeCYP51B* gene and the ORFs encoding wild-type or mutated TmeCYP51B proteins (ORF*) were subcloned into the pAg1-*TinCYP51B*/T upstream (SpeI/ApaI) of the *hph* cassette, respectively (Table 2). (D) Southern blotting. Aliquots of approximately 10 μg of genomic DNA from each strain were digested with XhoI or SphI and separated by electrophoresis on 0.8% (wt/vol) agarose gels. Lane 1, 1062Av1401 (parent strain); lane 2, ΔTmeCYP51B-14 (*TmeCYP51B* disruptant); lane 3, wTmeCYP51B/C-1 (revertant harboring the wild-type *TmeCYP51B* gene); lane 4, mTmeCYP51B/C-25 (mutant harboring the mutated *TmeCYP51B* gene). A fragment of about 480 bp of the *TmeCYP51B* locus was amplified by PCR with a pair of the primers P57 and P36 (Table S1) and used as a hybridization probe. DNA standard fragment sizes are shown on the left. (E and F) Evaluation of ITC and VRC susceptibility in the four T. mentagrophytes strains (1062Av1401, ΔTmeCYP51B-14, wTmeCYP51B/C-1, and mTmeCYP51B/C-25) by serial dilution drug susceptibility assays (E) and Etest assays (F). For serial dilution drug susceptibility assays, spores from each strain were spotted at different dilutions on SDA plates, as described in Materials and Methods. The plates were incubated at 28°C for 3 to 5 days (serial dilution drug susceptibility assays) or 4 days (Etest assays).

To determine which mechanism is involved in the resistance of the selected strains to azole compounds, we first looked for the presence of possible point mutations in the *TinCYP51A* and *TinCYP51B* genes, both encoding sterol 14α-demethylase, a target of azoles. Sequencing of *TinCYP51A* and *TinCYP51B* in all strains revealed that, in the three resistant strains, TIMM20116, TIMM20118, and TIMM20119, *TinCYP51B* contained a missense G/A, generating a Gly443Glu amino acid substitution in the encoded protein. We examined whether this single amino acid substitution was involved in the resistant phenotypes of these three strains. The point mutation generating the Gly443Glu substitution in the *TinCYP51B* protein was introduced into the endogenous *CYP51B* (*TmeCYP51B*) gene of an azole-susceptible dermatophyte strain by genetic manipulations. To enhance the generation of such a mutation, T. mentagrophytes (formerly Arthroderma vanbreuseghemii) was used as a recipient organism, because this species is genetically closely related to *T. indotineae* and because a variety of more efficient genetic manipulation tools have been developed in this species (1, 20, 21). The point mutation generating the Gly443Glu substitution was introduced into *TmeCYP51B* of T. mentagrophytes strain 1062Av1401, according to a gene replacement strategy with Agrobacterium tumefaciens-mediated transformation (ATMT) (Fig. 2A to C). The gene replacement of *CYP51B* with a Gly443Glu allele in T. mentagrophytes 1062Av1401 did not affect the ITC or VRC MICs, suggesting that the Gly443Glu substitution was not responsible for the resistance to azoles of these strains (Fig. 2E and F).

### *TinCYP51B* and *TinMDR3* are highly overexpressed in *T. indotineae* strains with low susceptibility to azole compounds.

**FIG 3:**
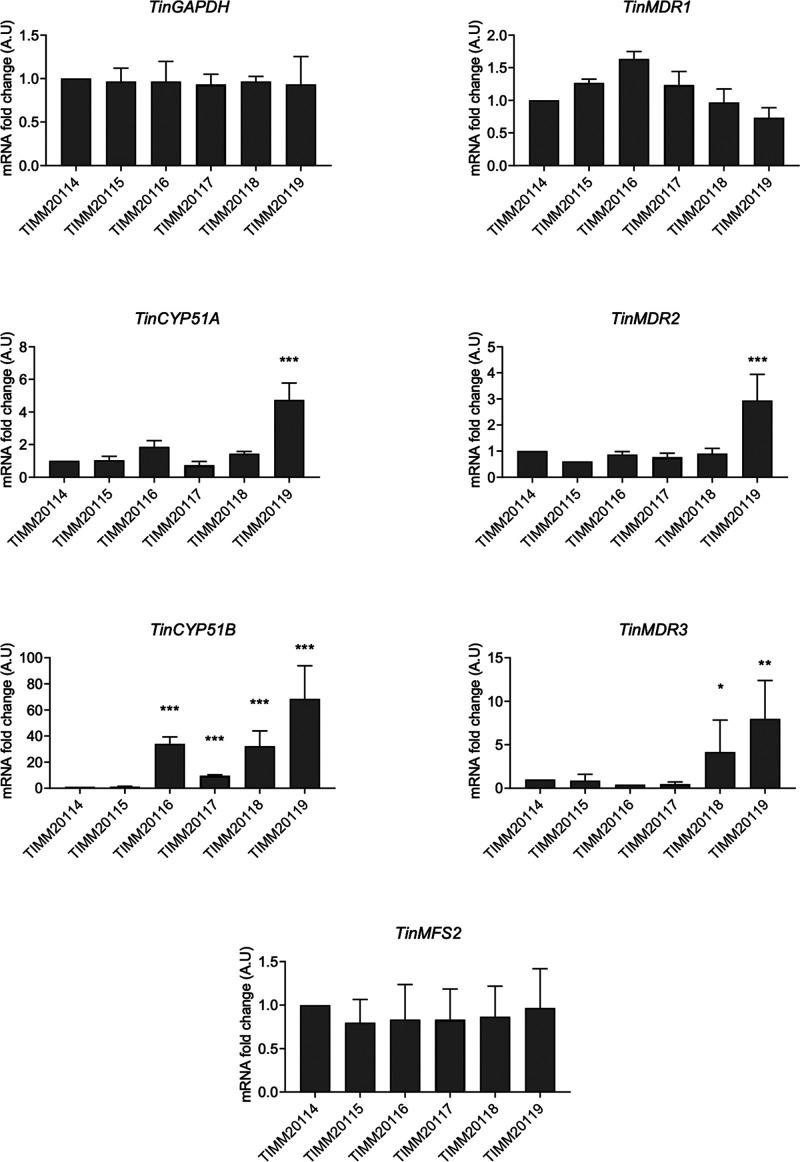
Expression levels of *CYP51A*, *CYP51B*, and 4 drug efflux transporter genes in six *T. indotineae* isolates, as determined by qRT-PCR. The fold change represents the level of gene expression compared with that of *T. indotineae* TIMM20114. The bars represent the standard deviation of the data obtained from three independent experiments. *, *P < *0.05; **, *P < *0.01; ***, *P < *0.001.

The expression of *TinCYP51A* and *TinCYP51B* encoding targets of azole compounds and those of four ITC and/or VRC transporter genes (*TinMDR1*, *TinMDR2*, *TinMDR3*, and *TinMFS2*) for which orthologs have been identified in T. rubrum were compared by quantitative real-time reverse transcription-PCR (qRT-PCR) between susceptible and low-susceptibility strains. *TruMDR2* and *TruMDR3* were involved in azole resistance in a strain of T. rubrum (6). *TruMDR1* was only found to be potentially involved in fungal cellular detoxification (22), but its orthologs in pathogenic fungi are drug efflux pumps (23–25). *TruMFS2* was found to be highly overexpressed in an azole-resistant strain of T. rubrum (K. Salamin and M. Monod, unpublished results). The primers used (see Table S1 in the supplemental material) were designed based on the deposited sequences for T. interdigitale, which is the species most closely related to *T. indotineae* (26). The most striking finding was the high expression of *TinCYP51B* in the four strains with low susceptibility to ITC and VRC (TIMM20116 to TIMM20119) compared with its expression in the susceptible strains TIMM20114 and TIMM20115, by a factor of 10 for TIMM20117 and up to 80 for TIMM20119 (Fig. 3). The expression of *CYP51A* was also significantly increased in TIMM20119, but only by a factor of 5 compared with its expression in the five other strains.

With respect to the transporters tested, the expression of *TinMDR3* in TIMM20118 and TIMM20119 was 5- and 8-fold higher, respectively, than in TIMM20114 and TIMM20115. In contrast, the expression of *TinMDR3* in the low-susceptibility strains TIMM20116 and TIMM20117 was comparable to that in the susceptible strains TIMM20114 and TIMM20115. The expression of *TinMDR2* was also 3 times higher in TIMM20119 than in the five other strains. The expression levels of the other transporter genes, *TinMDR1* and *TinMFS2*, in the four low-susceptibility strains were not higher than those in the susceptible strains TIMM20114 and TIMM20115. To summarize, *TinCYP51B* is highly overexpressed in all four susceptible strains, whereas *TinMDR3* shows a relatively high expression in TIMM20118 and TIMM20119. TIMM20119 also shows a smaller but significant increase in expression of *TinMDR2* and *TinCYP51A*.

### Disruption of *TinMDR3* in TIMM20118 and TIMM20119 does not attenuate ITC and VRC resistance.

**FIG 4:**
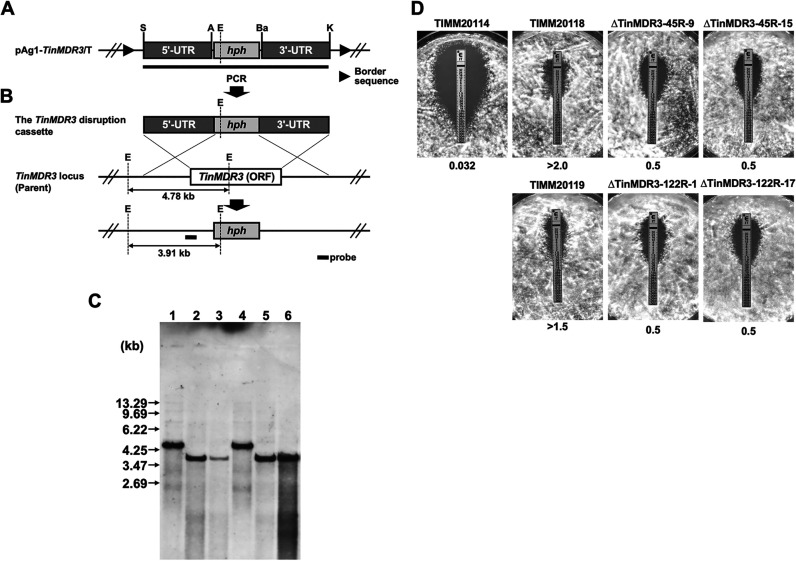
Disruption of the *MDR3* (*TinMDR3*) gene of *T. indotineae* TIMM20118 and TIMM20119 by gene replacement strategy. (A) Schematic representation of a part of the *TinMDR3*-targeting vector pAg1-*TinMDR3*/T. The *hph* cassette is composed of Aspergillus nidulans *trpC* promoter (*PtrpC*), E. coli hygromycin B phosphotransferase gene (*hph*), and A. fumigatus *cgrA* terminator (*TcgrA*). Border sequences are the specific regions that delineate the DNA to be transferred during Agrobacterium tumefaciens-mediated transformation. Restriction enzyme site: A, ApaI; Ba, BamHI; E, EcoRI; K, KpnI; P, PstI; S, SpeI. (B) Schematic representation of the *TinMDR3* locus before and after homologous recombination. (C) Southern blotting. Aliquots of approximately 10 μg of genomic DNA from each strain were digested with EcoRI and separated by electrophoresis on 0.8% (wt/vol) agarose gels. Lanes 1 to 6, TIMM20118 (parent strain), ΔTinMDR3-45MM-9, ΔTinMDR3-45MM-15, TIMM20119 (parent strain), ΔTinMDR3-122MM-1, and ΔTinMDR3-122MM-17, respectively. A fragment of about 530 bp of the *TinMDR3* locus in TIMM20119 was amplified by PCR with a pair of the primers P47 and P48 (Table S1) and used as a hybridization probe. DNA standard fragment sizes are shown on the left. (D) Evaluation of ITC susceptibility in the *TinMDR3* disruptants produced from TIMM20118 and TIMM20119, by Etest assays. The plates were incubated at 28°C for 4 days.

We examined the significance of *TinMDR3* overexpression in TIMM20118 and TIMM20119 on azole resistance by targeted gene disruption (Fig. 4). The protoplast/polyethylene glycol (PEG)-mediated transformation of TIMM20118 and TIMM20119 using the *TinMDR3* disruption cassette (Fig. 4A and B) resulted in the successful production of hygromycin B-resistant colonies on selective medium, 17 and 31 of which were chosen at random, respectively, and analyzed by molecular biological methods. Southern blotting analyses suggested disruption of the *TinMDR3* locus in four transformants (ΔTinMDR3-45R-9 and ΔTinMDR3-45R-15 from TIMM20118; ΔTinMDR3-122R-1 and ΔTinMDR3-122R-17 from TIMM20119) (Fig. 4C). The ITC and VRC susceptibilities of the four *TinMDR3* disruptants were evaluated with Etest assays, serial dilution drug susceptibility assays on Sabouraud dextrose agar (SDA) plates, and the Clinical and Laboratory Standards Institute (CLSI) broth microdilution method. Using ITC Etests, small differences in susceptibility were observed between *TinMDR3* mutants and the parental strains (Fig. 4D). VRC sensitivity could not be tested with Etests, because all strains were resistant to the highest concentration of VRC on the strips. No differences could be observed with serial dilution drug susceptibility assays on SDA plates and with the broth dilution method used to measure MICs (data not shown).

### *TinCYP51B* silencing increases susceptibility of *T. indotineae* to azole compounds.

To examine the importance of *TinCYP51B* overexpression in azole resistance, we tried to disrupt the *TinCYP51B* locus in TIMM20116, TIMM20117, TIMM20118, and TIMM20119 with a gene replacement strategy using ATMT with pAg1-*TinCYP51B*/T and/or the protoplast/PEG method with the *TinCYP51B* disruption cassette (Fig. S1). However, all attempts to disrupt *TinCYP51B* were unsuccessful (data not shown). Nevertheless, disruption could be carried out in T. mentagrophytes 1062Av1401 by using ATMT with the same plasmid, which revealed that *CYP51B* is not vital in this species. Following these results, we used RNA interference (RNAi) as an alternative method for *TinCYP51B* silencing in *TinCYP51B*-overexpressing strains with low sensitivity to azole compounds. RNAi is based on a natural phenomenon by which a double-stranded RNA (dsRNA) induces enzymatic degradation of mRNA in a sequence-specific manner.

**FIG 5:**
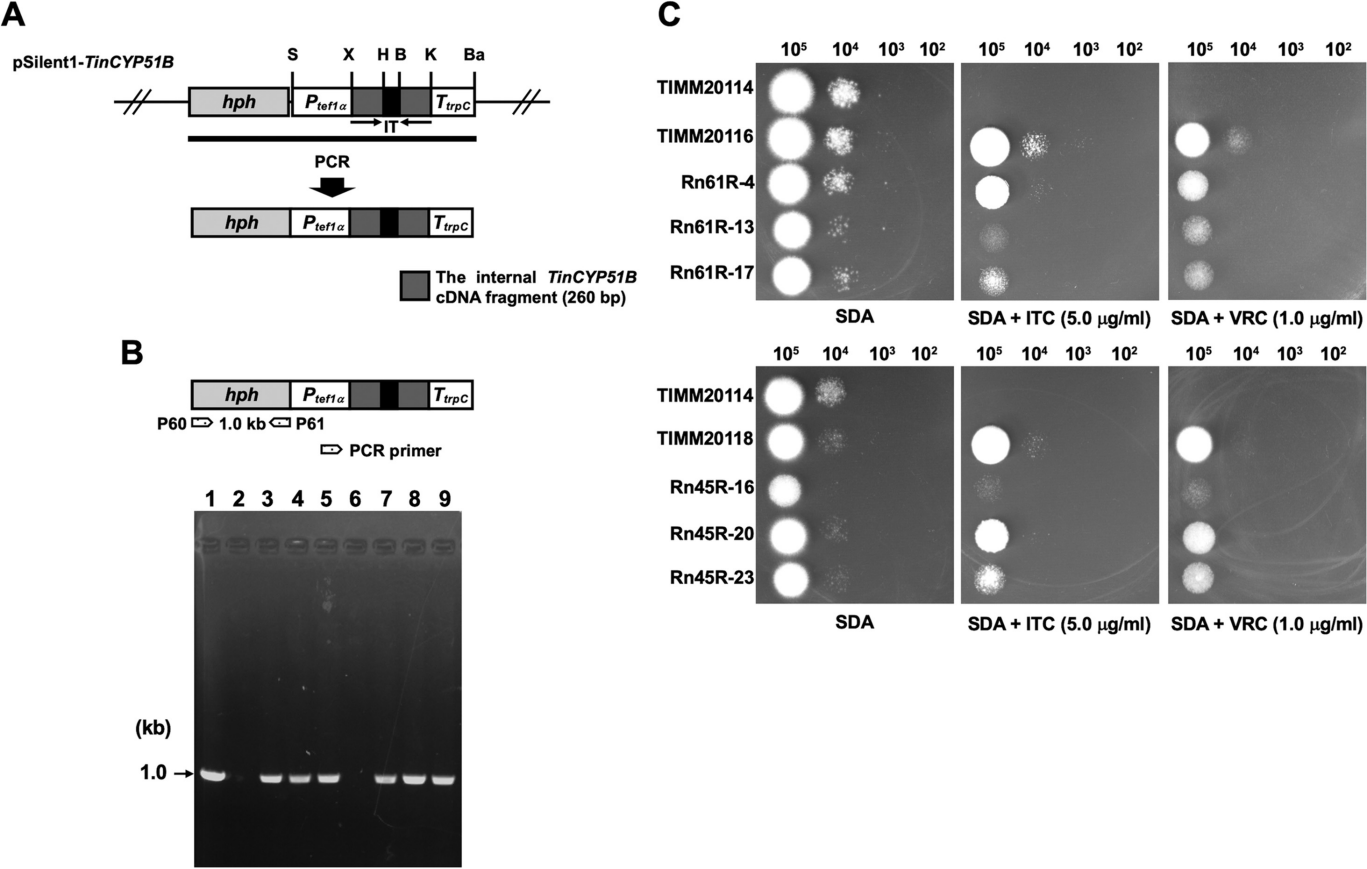
RNAi-mediated downregulation of *TinCYP51B* in the low azole-susceptibility *T. indotineae* strains TIMM20116 and TIMM20118. (A) Schematic representation of the construct expressing *TinCYP51B* hairpin RNA in the gene-silencing vector pSilent1-*TinCYP51B*. Arrows indicate the direction of the *TinCYP51B* cDNA. The *hph* cassette is composed of *PtrpC*, *hph*, and Aspergillus nidulans *trpC* terminator (*TtrpC*). Plasmid DNA of pSilent1-*TinCYP51B* was used as a template for PCR, to amplify the sequence indicated by the thick line. *Ptef1α*, the promoter sequence of *T. indotineae* translation elongation factor 1-α (*TinTef1α*) gene. IT, the M. oryzae *cut1* gene intron spacer. Restriction enzyme site: Ba, BamHI; B, BglII; K, KpnI; S, SpeI; X, XhoI. (B) PCR analysis of the short hairpin RNA (shRNA) clones. Genomic DNA samples from each strain were subjected to PCR with a pair of the primers P62 and P63 for amplification of the E. coli *hph* gene. Lanes 1 to 9, pSilent1-*TinCYP51B*, TIMM20116 (parent strain), the shRNA clone Rn61R-4, Rn61R-13, Rn61R-17, TIMM20118 (parent strain), Rn45R-16, Rn45R-20, and Rn45R-23, respectively. A DNA standard fragment size is shown on the left. (C) Evaluation of ITC and VRC susceptibility in the shRNA clones against the *TinCYP51B* gene by serial dilution drug susceptibility assays. Spores from each strain were spotted at different dilutions on SDA plates, as described in Materials and Methods. The plates were incubated at 28°C for 3 to 5 days.

Gene silencing in *T. indotineae* was carried out by transforming the fungus with an integrative hairpin RNA expression construct derived from the gene-silencing vector pSilent-1 (27), which was previously developed for ascomycete fungi. pSilent-1 carries a hygromycin B resistance cassette and a transcriptional unit for hairpin RNA expression with a spacer of the cutinase (*cut1*) gene intron 2 from the rice blast fungus Magnaporthe oryzae. pSilent1-*TinCYP51B* has duplicate sequences of the internal *TinCYP51B* cDNA (260 bp) cloned as inverted repeats separated by the *cut1* gene intron spacer of M. oryzae (Fig. 5A, Table 2).

**TABLE 2:** Plasmids used in this study

Plasmid	Description	Source or reference
pAg1	Streamlined version of the binary vector pBIN19 containing sequences necessary for replication in E. coli and Agrobacterium tumefaciens (*oriV* and *trfA*), E. coli neomycin phosphotransferase gene (*nptII*), and the transferable DNA (T-DNA) region, with a multiple cloning site within the T-DNA region	49
pAg1-*hph2*	The *hph* cassette (the promoter sequence of Aspergillus nidulans tryptophan C [*trpC*] gene [*PtrpC*] [GenBank accession no. X02390], E. coli hygromycin B phosphotransferase gene [*hph*], the termination sequence of Aspergillus fumigatus *cgrA* gene [*TcgrA*] [GenBank accession no. EAL84894])	This study
pAg1-*nptII*	The *nptII* cassette (*PtrpC*, *nptII*, *TcgrA*)	This study
pAg1-*TinCyp51B*/OE	The *nptII* cassette, the upstream region of *T. indotineae* translation elongation factor 1-α (*TinTef1 α*) gene (*Ptef1α*) (GenBank accession no. OK513035), *T. indotineae* CYP51B (*TinCYP51B*) cDNA, *TcgrA*	This study
pAg1-*TinCyp51B*/T	The 5′ UTR of *TinCYP51B* gene (2.52 kb) (GenBank accession no. OK539858), the *nptII* cassette, the 3′ UTR of *TinCYP51B* gene (2.51 kb)	This study
pAg1-wTmeCyp51B/C	The fragment TmeCYP51B1 (the 5′ UTR and wild-type ORF^*a*^ of *TmeCYP51B* gene) (3.59 kb), the *hph* cassette (*PtrpC*, *hph*, *TcgrA*), the 3′ UTR of *TinCYP51B* gene (2.51 kb)	This study
pAg1-*mTmeCyp51B*/C	The fragment TmeCYP51B2 (the 5′ UTR and mutated ORF of *TmeCYP51B* gene having a point mutation leading to the Gly443Glu substitution) (3.59 kb), the *hph* cassette (*PtrpC*, *hph*, *TcgrA*), the 3′ UTR of *TinCYP51B* gene (2.51 kb)	This study
pAg1-*TinMDR3*/T	The 5′ UTR of *TinMDR3* gene (2.60 kb) (GenBank accession no. OK539858), the *hph* cassette, the 3′ UTR of *TinMDR3* gene (2.51 kb)	This study
pSilent-1	The *hph* cassette (*PtrpC*, *hph*, the terminator sequence of A. nidulans *trpC* gene [*TtrpC*]), *PtrpC*, the intron 2 of Magnaporthe oryzae cutinase gene (*cut1*) (GenBank accession no. X61500), *TtrpC*	27
pSilent1-*TinCyp51B*	The *hph* cassette (*PtrpC*, *hph*, *TtrpC*), *Ptef1* α, the internal sense *TinCYP51B* cDNA fragment (0.26 kb), the intron 2 of M. oryzae *cut1* gene, the internal antisense *TinCYP51 B* cDNA fragment (0.26 kb), *TtrpC*	This study

a ORF, open reading frame.

The protoplast/PEG-mediated transformation of TIMM20116 and TIMM20118 using the *TinCYP51B* silencing cassette indicated in Fig. 5A (bold line) resulted in the successful production of hygromycin B-resistant colonies on selective medium, 25 and 20 of which were chosen at random, respectively, and tested for their growth properties on SDA supplemented with 2.0 μg/mL ITC. Six transformants (Rn61R-4, Rn61R-13, and Rn61R-17 from TIMM20116; Rn45R-16, Rn45R-20, and Rn45R-23 from TIMM20118) showed significant growth inhibition compared with their parental strains (data not shown). After a molecular biological analysis by PCR (Fig. 5B), the ITC and VRC susceptibilities of these transformants were evaluated by serial dilution drug susceptibility assays on SDA plates, which indicated that the susceptibilities had increased due to the RNAi-mediated downregulation of *TinCYP51B* (Fig. 5C). The ITC and VRC MICs of six transformants from TIMM20116 and TIMM20118 were also measured using the CLSI broth microdilution method (Table 3). RNAi-mediated downregulation of *TinCYP51B* in these transformants resulted in either no reduction or up to a 4-fold reduction in the MICs of ITC and VRC compared with their parental strains. As expected, qRT-PCR analysis revealed that the amount of *TinCYP51B* RNA was significantly decreased in the transformants compared with the parental strains (Table 3).

**TABLE 3:** Susceptibilities to ITC and VRC of *T. indotineae TinCYP51B* RNAi transformants and corresponding expression levels

*T. indotineae* sp. and strain	ITC MIC_80_ (μg/mL)	VRC MIC_80_ (μg/mL)	Fold expression of *TinCYP51B* (mean ± SD)^*a*^
TIMM20114 (control)	0.06	0.015	1
TIMM20116 (parent)	1.0	1.0	34.0 ± 5.3
Rn61R-4	0.5	0.25	12.6 ± 3.1
Rn61R-13	0.25	0.25	6.7 ± 0.9
Rn61R-17	0.5	0.25	12.4 ± 2.9
TIMM20118 (parent)	1.0	1.0	35.0 ± 12.1
Rn45R-16	0.5	0.25	5.7 ± 1.4
Rn45R-20	1.0	0.5	14.1 ± 5.3
Rn45R-23	1.0	0.5	17.8 ± 7.3

a Results represent expression levels from three independent real-time PCR experiments. Expression levels of *TinCYP51B* genes were indicated as relative fold changes compared to the CT mean of the data from TIMM20114.

### Overexpressing *TinCYP51B* confers resistance to the azole-susceptible strain TIMM20114 of *T. indotineae*.

**FIG 6:**
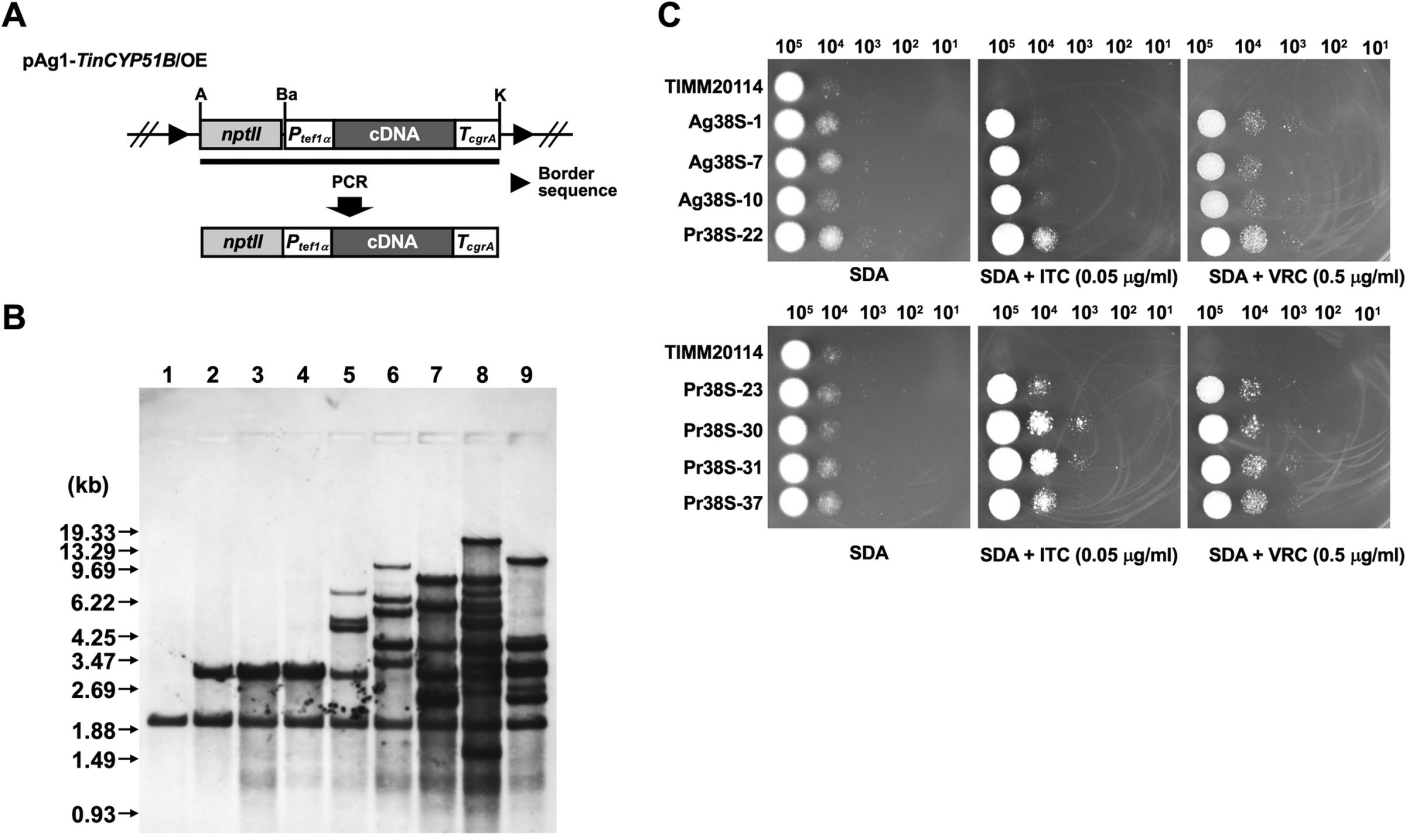
Production of *T. indotineae* transformants overexpressing the *TinCYP51B* cDNA. (A) Schematic representation of a part of the *TinCYP51B* expression vector pAg1-*TinCYP51B*/OE. The *nptII* cassette is composed of *PtrpC*, E. coli neomycin phosphotransferase gene (*nptII*), and *TtrpC*. Plasmid DNA of pAg1-*TinCYP51B*/OE was used as a template for PCR, to amplify the *TinCYP51B* overexpression cassette indicated by the thick line. *Ptef1α*, the promoter sequence of *T. indotineae* translation elongation factor 1-α (*TinTef1α*) gene. Border sequences are the specific regions that delineate the DNA to be transferred during Agrobacterium tumefaciens-mediated transformation. Restriction enzyme site: A, ApaI; Ba, BamHI; K, KpnI. (B) Southern blotting. Aliquots of approximately 10 μg of total DNA from each strain were digested with XhoI and separated by electrophoresis on 0.8% (wt/vol) agarose gels. Lanes 1 to 9, TIMM20114 (parent) and the *TinCYP51B*-overexpressing clones Ag38S-1, Ag38S-7, Ag38S-10, Pr38S-22, Pr38S-23, Pr38S-30, Pr38S-31, and Pr38S-37, respectively. An internal fragment (about 260 bp) of the *TinCYP51B* cDNA was amplified by PCR with a pair of the primers P58 and P59 (Table S1) and used as a hybridization probe. DNA standard fragment sizes are shown on the left. (C) Evaluation of ITC and VRC susceptibility in the *TinCYP51B*-overexpressing clones by serial dilution drug susceptibility assays. Spores from each strain were spotted at different dilutions on SDA plates, as described in Materials and Methods. The plates were incubated at 28°C for 3 to 5 days.

To evaluate the role of *TinCYP51B* overexpression in the *T. indotineae* strains with low susceptibility to azole compounds, we overexpressed *TinCYP51B* cDNA in the azole-susceptible strain TIMM20114. Genetic transformation of TIMM20114 by ATMT with the *TinCYP51B* overexpression construct pAg1-*TinCYP51B*/OE or by the protoplast/PEG-mediated method with the *TinCYP51B* overexpression cassette (Fig. 6A) each resulted in the successful production of more than 30 G418-resistant colonies on selective medium, from which 20 each were chosen at random and tested for their growth properties on SDA supplemented with 0.1 μg/mL ITC. Three ATMT-mediated transformants (Ag38S-1, Ag38S-7, and Ag38S-10) and five protoplast/PEG-mediated transformants (Pr38S-22, Pr38S-23, Pr38S-30, Pr38S-31, and Pr38S-37) showed vigorous growth (data not shown), and their genomic DNA was extracted and analyzed by molecular biological methods. Southern blotting revealed that one or two ectopic copies of the *TinCYP51B* cDNA were inserted into the chromosomes of ATMT-mediated transformants (Fig. 6B, columns 2 to 4). A tandem insertion may have occurred in Ag38S-7 and Ag38S-10. The size of the new fragment is the same in all three ATMT transformants, but the intensity of the upper band in Ag38S-7 and Ag38S-10 appeared to be twice that of Ag38S-1 and twice that of the lower band in the three transformants. In contrast, more than two ectopic copies of *TinCYP51B* cDNA were inserted into the chromosomes of the protoplast/PEG-mediated transformants (Fig. 6B, columns 5 to 9). The ITC and VRC susceptibilities of these transformants were evaluated by serial dilution drug susceptibility assays on SDA plates, and the overexpression of *TinCYP51B* was shown to increase ITC and VRC resistance (Fig. 6C). The ITC and VRC MICs of these transformants were also measured using the CLSI broth microdilution method (Table 4). The ITC and VRC MICs of the five transformants that were generated by the protoplast/PEG method were about 4 to 8 times higher than those of the susceptible strain TIMM20114 (Table 4). The three transformants that were generated by ATMT showed lower ITC and VRC resistance and had a lower copy number of *TinCYP51B* cDNA than that of the transformants generated by the protoplast/PEG method.

**TABLE 4:** Susceptibilities to ITC and VRC of *T. indotineae* transformants overexpressing the *TinCYP51B* cDNA and corresponding expression levels

*T. indotineae* sp. and strain	ITC MIC_80_ (μg/mL)	VRC MIC_80_ (μg/mL)	Fold expression of *TinCYP51B* (mean ± SD)^*a*^
TIMM20114 (parent)	0.06	0.015	1
Ag38S-1	0.25	0.06	4.3 ± 0.6
Ag38S-7	0.125	0.03	9.3 ± 1.4
Ag38S-10	0.125	0.03	9.9 ± 1.3
Pr38S-22	0.25	0.06	11.5 ± 5.5
Pr38S-23	0.25	0.06	9.1 ± 0.8
Pr38S-30	0.5	0.125	30.0 ± 1.8
Pr38S-31	0.5	0.125	19.9 ± 3.1
Pr38S-37	0.5	0.06	18.1 ± 1.6

a Results represent expression levels from three independent real-time PCR experiments. Expression levels of *TinCYP51B* genes were indicated as relative fold changes compared to the CT mean of the data from TIMM20114.

### Genomes of *T. indotineae* strains with low susceptibility to azole compounds harbor multiple copies of *TinCYP51B*.

**FIG 7:**
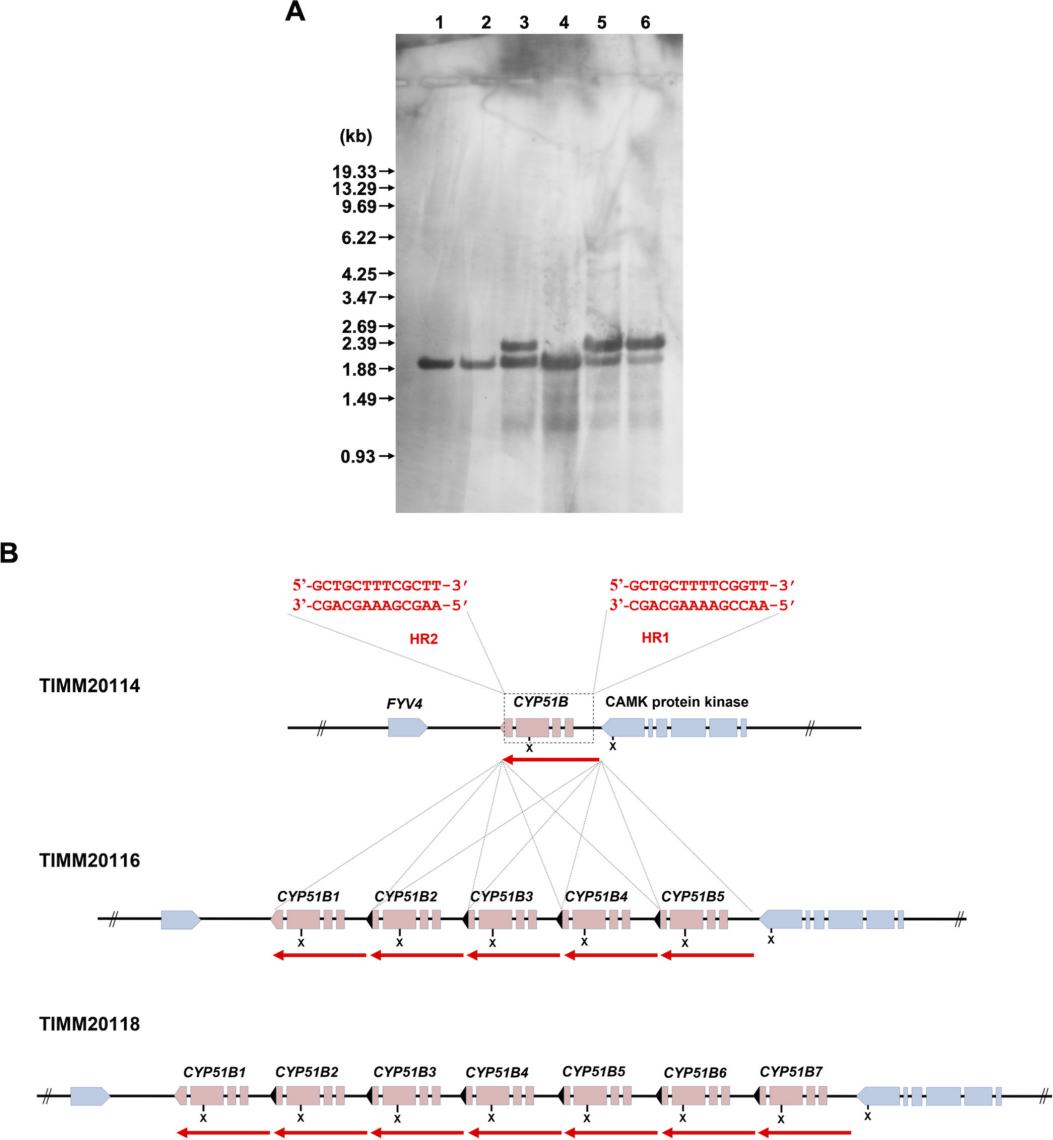
Organization of the *CYP51B* loci within the genomes of six *T. indotineae* strains (TIM20114, TIMM20115, TIMM20116, TIMM20117, TIMM20118, and TIMM20119). (A) Southern blotting. Aliquots of approximately 10 μg of total genomic DNA from each strain were digested with XhoI and separated by electrophoresis on 0.8% (wt/vol) agarose gels. Lanes 1 to 6, TIMM20114, TIMM20115, TIMM20116, TIMM20117, TIMM20118, and TIMM20119, respectively. An internal fragment (about 260 bp) of the *TinCYP51B* cDNA was amplified by PCR with a pair of the primers P58 and P59 (Table S1) and used as a hybridization probe. DNA standard fragment sizes are shown on the left. (B) Schematic representation of the organization of the *TinCYP51B* genes in the six strains. The *TinCYP51B* genes are indicated in red, with a red arrow showing the extent of the duplicated blocks. The truncated C terminus of the recombinant copies (*TinCYP51B2* to *TinCYP51B5* in TIMM20116 and TIMM20119, and *TinCYP51B2* to *TinCYP51B7* in TIMM20118) is marked in black. The genes marked in blue are the neighboring genes. The susceptible strain TIMM20114 and the resistant strain TIMM2117 both harbor one copy of the *TinCYP51B* gene, whereas both TIMM20116 and TIMM20119 have the same *TinCYP51B* loci architecture with 5 copies. The conserved homologous recombination sites HR1 and HR2 are described in red at the top of the figure. The XhoI restriction sites at the *TinCYP51B* loci are also indicated (X). The sequences of the TIMM20114 and TIMM20118 CYP51B loci can be found in the supplemental material.

The overexpression of *TinCYP51B* could be due to gene deregulation or to multiple copies in the genome. To test the latter possibility, we tried to estimate the copy number of this gene in the genomes of the six strains using Southern blotting of the genomic DNA cut with the XhoI restriction enzyme (Fig. 7A). Southern blotting revealed one *TinCYP51B* band for the susceptible strains TIMM20114 and TIMM20115 and the resistant strain TIMM20117. An additional band with a slightly higher molecular weight—and with a different intensity from one strain to another—was revealed for the strains TIMM20116, TIMM20118, and TIMM20119, which are resistant to azole compounds. These results, which were interpreted as the possible presence of several copies of *TinCYP51B* in some of the resistant strains, were corroborated by qRT-PCR experiments using genomic DNA as the target (data not shown). Both experiments strongly suggested that multiple copies of *TinCYP51B* were present in resistant strains, assuming that TIMM20114 and the other susceptible strain, TIMM20115, that was used as a control had one copy of *TinCYP51B*.

To confirm the existence of several *TinCYP51B* copies in *T. indotineae*-resistant strains, the whole genomes of the six strains were sequenced using the PacBio sequencing technique (Table S2). Analysis of the sequences revealed that the two susceptible strains TIMM20114 and TIMM20115, and the resistant strain TIMM20117, contained only one copy of *TinCYP51B*, whereas the three resistant strains TIMM20116, TIMM20118, and TIMM20119 contained 5, 7, and 5 copies, respectively (Fig. 7B, see also the supplemental material). In each locus, the original gene was named *TinCYP51B1*, and the additional copies were named *TinCYP51B2* to *TinCYP51B5* in TIMM20116 and TIMM20119, and *TinCYP51B2* to *TinCYP51B7* in TIMM20118. In general, the PacBio sequencing results agreed with those of the Southern blot experiments, except for TIMM20116, for which the top band in Fig. 7A was of comparable intensity to the bottom band, suggesting that a single extra copy was present in this strain. Southern blotting remains a more qualitative technique, whereas the results by single-molecule long sequencing using the PacBio technique are indisputable.

The duplicated regions comprised direct tandem repeats of 100% identical blocks of 2,404 bp, inserted at the 5′ end of the original *TinCYP51B* gene. These blocks included 631 bp of the original promoter and almost the entire coding region; only the last 5 codons were missing. At the borders of the original block were two almost identical sequences, 5′-AACCGAAAAGCAGC-3′ (HR1, for homologous recombination site 1) and 5′-AAGCGAAAGCAGC-3′ (HR2). HR1 was 644 to 630 nucleotides upstream of the *TinCYP51B* ATG, at the extreme end of the 3′ end of the neighbor gene Ca^2+^/calmodulin-dependent protein kinase (*CAMK*), and 156 nucleotides downstream of its predicted stop codon. HR2 was near the end of the coding sequence of *TinCYP51B* (27 to 14 nucleotides upstream of the stop codon). The presence of this conserved 5′-AAC/GCGAAA(A)GCAG-3′ consensus sequence at these positions apparently allowed homologous recombination, leading to amplification of a genomic region containing the promoter and almost the entire *TinCYP51B* coding region. These sequences also explained why the duplicated blocks were identical in independent resistant strains such as TIMM20116, TIMM20118, and TIMM20119. Each block contained an XhoI site, which explained the presence of the lowest electrophoretic mobility band for these three resistant strains in the Southern blot analysis (Fig. 7A). The size of this band corresponds to the separation of the XhoI sites within the tandem repeats, which equals the size of the duplicated blocks (2,404 bp). The band with the highest electrophoretic mobility in the Southern blot analysis corresponded to a fragment of 2,117 bp, in agreement with the location of the XhoI restriction sites in the *TinCYP51B* locus of these strains (Fig. 7A and B). Fortuitously, consecutive insertions of the blocks in direct tandems led to the expression of *TinCYP51B* proteins, which differ from the original *TinCYP51B* only in their C terminus, which contains a portion encoded by the 3′-untranslated region (UTR) of the nearby *CAMK* gene. This small truncation seemed not to impact the activity of the *TinCYP51B* copies, as suggested by the resulting resistance of those strains to azoles.

Due to synteny between dermatophytes, *CYP51B* and *CAMK* showed the same organization, and similar HR1 and HR2 sequences could be found in the same places in other dermatophytes (Table S3). This suggested that *CYP51B* amplification could occur in other dermatophytes, such as T. rubrum, and might also affect the susceptibility of these species to azoles.

## DISCUSSION

*TinCYP51B* encodes a cytochrome P450 sterol 14α-demethylase, which is inhibited by azole antifungals such as ITC and VRC. Sterol 14α-demethylases play a crucial role in ergosterol biosynthesis by catalyzing the oxidative removal of 14α-methyl groups from sterol precursors. We showed that the resistance of *T. indotineae* strains to azole compounds is mediated by overexpression of *TinCYP51B* due to multiple copies of this gene in tandem. Silencing of *TinCYP51B* by RNAi resulted in increased susceptibility to ITC and VRC. Overexpression of the gene encoding the drug target is an efficient mechanism for acquiring resistance, as it compensates for the effects of drugs by altering the balance in favor of the targets of the drugs.

Only small differences in susceptibility were observed between *TinMDR3* disruptants and parental strains using Etests. In contrast, MICs and serial dilution drug susceptibility tests on SDA agar plates revealed no differences in susceptibility. The role of *TinMDR3* overexpression in resistant *T. indotineae* strains could be masked by the effect of *TinCYP51B* overexpression. These results differed from those obtained with a clinical strain of T. rubrum (TIMM20092), where resistance to azole antifungals was only due to the overexpression of two transporter-encoding genes, *TruMDR2* and *TruMDR3* (6, 28). Disruption of these two genes rendered the fungus susceptible, like a susceptible strain of T. rubrum (TIMM 20112).

Several amino acid substitutions of *TinCYP51B* were recently identified in *T. indotineae* isolates, including Gly443Glu (29); however, the Gly443Glu substitution detected in *TinCYP51B* of strains TIMM20116, TIMM20118, and TIMM20119 did not appear to play a role in their low susceptibility to azoles (Fig. 2). It was also previously reported that *T. indotineae* strains containing an Ala448Thr substitution at the C terminus of the SQLE had higher ITC and VRC MICs, on average (7); however, strain TIMM20114 was susceptible to azole, despite the Ala448Thr substitution. Both mutations should, rather, be considered nucleotide polymorphisms in *T. indotineae*, not selected by azoles in the fungal environment.

### CYP51 isoforms in fungi.

The number of CYP51 isoforms varies from one fungal species to another. Saccharomyces cerevisiae contains only one CYP51 isoform (30, 31), whereas two isoforms, here called AfuCYP51A and AfuCYP51B, are present in the pathogenic fungus A. fumigatus (32), and three isoforms are present in Aspergillus oryzae and in the plant pathogen Fusarium graminearum (CYP51A, CYP51B, and CYP51C). The S. cerevisiae *CYP51/ERG11* mutant can be complemented by A. fumigatus *AfuCYP51A* and *AfuCYP51B* (33). F. graminearum CYP51B is the enzyme primarily responsible for sterol 14α-demethylation and plays an essential role in ascospore formation. CYP51A in the latter fungus is an additional sterol 14α-demethylase, induced on ergosterol depletion and responsible for the intrinsic variation in azole sensitivity. Meanwhile, F. graminearum CYP51C does not encode a sterol 14α-demethylase but is required for full virulence on host wheat ears (34, 35).

The ergosterol biosynthetic pathway differs in filamentous fungi, such as Aspergillus species, from that in yeast. The ring system of lanosterol in S. cerevisiae is first demethylated in three enzymatic steps, leading to the intermediate zymosterol, and a methyl group is then added to zymosterol by the sterol 24-C-methyltransferase, to form fecosterol. In Aspergillus spp., lanosterol is first transmethylated by the sterol 24-C-methyltransferase, leading to the intermediate eburicol, which is then demethylated in three steps to form fecosterol (36). Dermatophytes are closely related to A. fumigatus and, like this species, contain two isoforms, CYP51A and CYP51B. These two enzymes probably also function as eburicol demethylases. It should be noted that several isoforms of other ergosterol pathway enzymes, which are targets of antifungals, are also present in filamentous fungi. This is the case for beta-hydroxymethylglutarate reductase, which is targeted by statins such as lovastatin. Beta-hydroxymethylglutarate reductases are encoded by the *hmg1* and *hmg2* genes in A. fumigatus and by five genes in A. oryzae. Isoforms also exist for squalene synthase (targeted by squalestatin), SQLE (targeted by allylamines), C-14 reductase and C-8 sterol isomerase (both targeted by morpholines), and Δ24-sterol C-methyltransferase (targeted by tomatidine) (32, 37, 38).

### A new mechanism of drug resistance in fungi, with the amplification of the gene encoding the drug target.

Overexpression of the gene encoding the target of azole antifungals has been demonstrated with *AfuCYP51A* and *AfuCYP51B* in A. fumigatus. The mechanism of *AfuCYP51B* overexpression has not been studied (39); however, *AfuCYP51A* overexpression was found to be mediated by the presence of two copies of a 34-bp sequence in tandem in the *AfuCYP51A* promoter, together with the presence of an A for T substitution at position 364 in the gene-coding sequence. This nucleotide change led to the Leu98His substitution in the protein (13). In Candida glabrata, overexpression of *CYP51* was linked to an increase in copy number due to duplication of the entire chromosome containing the CYP51 gene (14). However, the low-susceptibility phenotype was unstable, and a gradual loss of the duplicated chromosome was seen in successive subcultures of the isolate in fluconazole-free medium.

Another mechanism is involved in the overexpression of the azole antifungal target in *T. indotineae*. Resistant strains contain multiple copies of *TinCYP51B* in tandem, with up to seven copies of the gene being found in strain TIMM20118. To our knowledge, this is the first time that such a mechanism has been described in the area of resistance acquisition toward azoles. Out of the four azole-resistant strains investigated in this study, three harbored tandem duplications of *TinCYP51B.* Only the resistant strain TIMM20117 contained a single *TinCYP51B* gene, whose overexpression could be explained by another mechanism, possibly a mutation in a transcription factor, as has been postulated for the overexpression of transmembrane transporters involved in azole resistance in clinical isolates of T. rubrum (6, 28). The lower overexpression of *TinCYP51B* in strain TIMM20117 compared with strains TIMM20116, TIMM20118, and TIMM20119 was consistent with the lower MICs toward ITC and VRC (Table 1).

Gene duplications within genomes can be classified into two types of cluster organization (40). The first, most frequent type includes genes sharing a significant level of identity in the amino acid sequences of their predicted protein product and organized in a similar manner (synteny) on different parts of the genome. The second cluster type is based on one gene unit tandemly repeated, and the level of nucleic acid identity is high within the coding sequence and the noncoding region between the two repeats. The *TinCYP51B* amplifications identified within the azole-resistant strains TIMM20116, TIMM20118, and TIMM20119 correspond to this second type of cluster organization. In addition to the coding sequences of the *TinCYP51B* tandems, the introns and intergenic regions are also 100% identical, suggesting that the gene amplification occurred very recently. The presence of two homologous sequences, HR1 and HR2—the first at the beginning of the promoter and the second near the end of the coding sequence—generates tandem blocks of *CYP51B*. Each block contains a copy of the *CYP51B* gene that is 100% identical to that of the susceptible strain TIMM20114, corresponding to the original gene, before the duplication events occurred.

Although we have described this mechanism for the first time in fungi, tandem genomic amplifications have been identified in strains of Streptococcus agalactiae, resulting in resistance to sulfonamides and trimethoprim (41, 42). The 4-fold amplification of 13.5 kb and the duplication of 92 kb leading, respectively, to sulfonamide and trimethoprim resistance showed different stabilities, the former being lost at a frequency of 0.003 per generation and the latter at a frequency of 0.035 per generation.

Low susceptibility of *T. indotineae* to azole compounds has been frequently reported. Low-susceptibility strains reached 25% in a large survey, but it is not clear whether these strains were generated during dermatophytosis treatment. It is likely that the tandem *TinCYP51B* repeated sequences found in the low-susceptibility strains of *T. indotineae* were generated under selective pressure, by long exposure to treatment, or in the environment in polluted soil, in a similar manner to that described for A. fumigatus. Human-to-human transmission can occur, because this species is anthropophilic, like T. interdigitale; both species are considered anthropophilic offshoots of the T. mentagrophytes species complex.

### Perspectives.

The discovery of the *TinCYP51B* tandem repeats leads to the question of their stability. As for S. agalactiae tandem repeats, it is reasonable to think that 100% identical tandem blocks might be targets for continual homologous recombination from one generation to another. The selection pressure by azoles is the reason for the maintenance of several copies of the *TinCYP51B* gene in the low-susceptibility strains. However, long-read sequencing has provided a picture of the situation at a precise moment of evolution. The dynamics of these regions will be followed in the future. Southern blot analysis, as performed in this study, provides an interesting tool to check the evolution of the size of those regions in strains TIMM20116, TIMM20118, and TIMM20119 but can also be used as an efficient tool to identify additional *TinCYP51B* amplifications in other low-susceptibility strains. The fortuitous presence of the conserved HR1 and HR2 sequences at just the right positions explains the possible amplification of *TinCYP51B* and the acquisition of low-susceptibility *T. indotineae* strains. Such a mechanism may not be limited to *T. indotineae*, because HR1 and HR2 are also localized in conserved regions of the genome of other dermatophyte species (Table S3), a phenomenon that would also allow amplification of the *CYP51B* gene in these fungi. This could explain the decreased susceptibility of other dermatophyte strains, such as T. rubrum clinical strains, toward azoles. In Microsporum canis, which is phylogenetically more distant, a sequence similar to HR2, but not HR1, can still be found. A. fumigatus also shows the same organization of the orthologs of *CYP51B* (Afu7g03740) and the *CAMK* gene (Afu7g03750), but we did not find any consensus sequences that would enable amplification of A. fumigatus *CYP51B*.

## MATERIALS AND METHODS

### Strains and growth media.

Six *T. indotineae* strains—TIMM20114 (UKJ1676/17; IFM 67092), TIMM20115 (UKJ1700/17II; IFM 67093), TIMM20116 (UKJ1708/17; IFM 67094), TIMM20117 (200087/18; IFM 67095), TIMM20118 (UKJ1687/17; IFM 67096), and TIMM20119 (200123/18; IFM 67097), which are listed in Table 1—were collected in India and previously tested for azole susceptibility (7). Glycerol stocks (15%; vol/vol) of these fungi, which were stored at −80°C, were used for conventional culture on SDA and Sabouraud dextrose broth (SDB) (Bio-Rad) at 28 to 30°C. T. mentagrophytes (formerly *Arthroderma vanbreuseghemii*) 1062Av1401 (21), which lacks a homolog of the human gene *XRCC5*, which encodes Ku80 (43), was used as a host strain for the production of T. mentagrophytes *CYP51B* (*TmeCYP51B*)-lacking mutants. Spore formation was promoted at 28°C using 1/10 SDA (0.1% [wt/vol] Bacto peptone [BD Biosciences], 0.2% [wt/vol] dextrose, 2% [wt/vol] agar) supplemented with 500 μg/mL cycloheximide and 50 μg/mL chloramphenicol (Wako Pure Chemical). A. tumefaciens EHA105 was maintained as previously described (44). Escherichia coli DH5α (Nippon Gene) was used for molecular cloning.

### Spore stock suspensions.

*T. indotineae* and T. mentagrophytes spores were collected from cultures on 1/10 SDA using sterile swabs and suspended in 3 mL sterile distilled water (dH_2_O). To obtain standardized spore stock suspensions, optical density (OD) values were determined at a wavelength of 600 nm (GeneQuant 1300 Spectrophotometer, Biochrom) and diluted to a value of 1.0; when considering the stock suspensions, an OD value of 1.0 was found to correspond to 2.2 × 10^7^ to 2.3 × 10^7^ CFU/mL.

### Chemicals.

ITC (Wako Pure Chemical) and VRC (Bio-Techne) were dissolved in dimethyl sulfoxide (DMSO) (Wako Pure Chemical) to constitute stock solutions (1.0 or 10 mg/mL for less soluble compounds). Stock solutions were stored at −20°C until use.

### Drug susceptibility assays for *T. indotineae*.

Using spore suspension stocks, MICs were determined according to guidelines for the broth microdilution method of the CLSI (45), except for the use of SDB instead of RPMI 1640 medium, if necessary. After incubation, the plates were visually evaluated and also read at 595 nm using a microtitration plate spectrophotometer (Multiskan Ascent, Thermo Fisher Scientific). The MIC_80_ was defined as the lowest concentration of the drug present in the wells showing growth inhibition of ≥80% compared with absorbance values obtained without antifungal agents.

For serial dilution drug susceptibility assays on agar plates, aliquots of 10 μL of 10-fold serial dilutions of the conidial suspensions, which contained 1 × 10^5^ to 1 × 10^1^ cells, were spotted on SDA containing the desired concentration of ITC or VRC. The dishes were incubated at 28°C for 3 to 5 days.

Etests were performed as previously described (28).

### *T. indotineae Cyp51A* and *Cyp51B* sequencing.

Genomic DNA of each *T. indotineae* strain was extracted from the growing mycelia using the DNeasy plant minikit (Qiagen) according to the manufacturer’s protocol. DNA fragments encoding *T. indotineae Cyp51A* (*TinCYP51A*) and *Cyp51B* (*TinCYP51B*) were amplified by PCR with a standard protocol using P1-P2 and P3-P4 primers, respectively (Table S1), and 200 ng of *T. indotineae* genomic DNA. DNA sequencing was performed by Microsynth (Switzerland).

### qRT-PCR analyses.

Total RNA extraction and cDNA synthesis for qRT-PCR were performed as described previously for T. rubrum (6). *T. indotineae* strains were grown in 50 mL SDB in 500-mL tissue culture flasks. Plugs from fresh fungal cultures were used as inoculates. The liquid cultures were carried out at 30°C without shaking. After 7 days, the growing mycelia from each strain were collected, frozen, and ground under liquid nitrogen. Total RNA was extracted using the RNeasy plant minikit (Qiagen) and then treated with DNase I (Qiagen). First-strand cDNA was synthesized using a high-capacity RNA-to-cDNA kit (Thermo Fisher Scientific). The qRT-PCR analysis was performed using Power SYBR green PCR master mix on a StepOne real-time PCR system (Thermo Fisher Scientific) under standard conditions, according to the manufacturer’s recommendations, with cDNA or genomic DNA as a target.

The primers used to amplify *TinACT*, *TinGAPDH*, *TinMDR1*, *TinMDR2*, *TinMDR3*, *TinMFS2*, *TinCYP51A*, and *TinCYP51B* are listed in Table S1. Expression levels of the genes encoding transporters were examined as relative fold changes compared with their levels in the strain TIMM20114, which was deemed susceptible to azoles. The relative quantification of gene expression was calculated according to the 2^ΔΔ^*^CT^* method (where *CT* is the threshold cycle). The statistical significance of the expression levels of target genes among strains was evaluated using Student’s *t* test.

### Genome sequencing and assembly.

The whole-genome sequencing and data analysis of *T. indotineae* strains were performed by Bioengineering Lab. Co., Ltd. (Japan). Genomic DNA was extracted from the growing mycelia according to a method described by Girardin et al. (46), with several minor modifications. After elimination of short DNA fragments (<10 kb) using the short read eliminator XS kit (Circulomics), the resulting DNA was sheared to 10 to 20 kb on the g-TUBE device (Covaris) prior to library preparation. High-fidelity (HiFi) sequencing libraries were prepared using the SMRTbell Express template prep kit 2.0 (Pacific Biosciences) and bound to the sequencing polymerase enzyme using the Sequel II binding kit 2.0 (Pacific Biosciences) according to the manufacturer’s protocol. Shotgun genomic DNA sequence data were collected on the Sequel IIe system (Pacific Biosciences) and assembled using the IPA HiFi genome assembler (version 1.5.0) (Pacific Biosciences).

### Construction of plasmid vectors for genetic manipulation in *T. indotineae*.

For overexpression of *TinCYP51B* in the azole-susceptible *T. indotineae* strain TIMM20114, a binary vector pAg1-*TinCYP51B*/OE was constructed as follows. The promoter sequence of the *T. indotineae* translation elongation factor 1-α (*TinTef1α*) gene (*Ptef1α*) was amplified from genomic DNA of *T. indotineae* strain TIMM20119 with the P21-P22 primers, and the terminator of the A. fumigatus *cgrA* gene (*TcgrA*) was amplified from the binary vector pAg1-*nptII* (Table 2) with the P23-P24 primers. The *TinCYP51B* cDNA was prepared from total RNA of *T. indotineae* strain TIMM20119 by reverse transcription (RT)-PCR with the PrimeScript one-step RT-PCR kit (version 2); (TaKaRa Bio) and the P25-P26 primers. The three obtained fragments were fused by overlap extension PCR with the P21-P24 primers, resulting in generation of the *TinCYP51B* cDNA cassette. This cDNA cassette was digested with BamHI/KpnI and cloned into the BamHI-KpnI sites of pAg1-*nptII*, resulting in generation of pAg1-*TinCYP51B*/OE.

To construct the plasmid vector pSilent1-*TinCYP51B* for RNAi-mediated downregulation of *TinCYP51B* in low-susceptibility *T. indotineae* strains TIMM20116 and TIMM20118, short internal sense and antisense *TinCyp51B* cDNA fragments were amplified with the P27-P28 and P29-P30 primers, respectively. The resulting two fragments were digested with XhoI/HindIII and BglII/KpnI, respectively, and cloned into the XhoI-HindIII and BglII-KpnI sites of the gene silencing vector pSilent-1 (GenBank accession no. LT827033) (Table 2) (27). *Ptef1α* was amplified from the *TinCYP51B* cDNA cassette with the P31-P34 primers, digested with SpeI/XhoI, and cloned into the SpeI/XhoI double-digested pSilent-1, resulting in generation of pSilent1-*TinCYP51B*.

To construct the *TinCYP51B*-targeting vector pAg1-*TinCYP51B*/T and the *TinMDR3-*targeting vector pAg1-*TinMDR3*/T (Table 2), approximately 2.5 to 2.6 kb of the upstream and downstream fragments of *TinCYP51B* and *TinMDR3*, respectively, were amplified from genomic DNA of *T. indotineae* strain TIMM20119 with the P35-P36 and P37-P42 primers and the P43-P48 and P49-P52 primers, respectively. The resulting upstream and downstream fragments of *TinCYP51B* and *TinMDR3* were digested with SpeI/ApaI and BamHI/KpnI, respectively, and cloned into the SpeI-ApaI and BamHI-KpnI sites of pAg1-*nptII* (for *TinCYP51B*) or pAg1-*hph2* (for *TinMDR3*) (Table 2) to generate pAg1-*TinCYP51B*/T and pAg1-*TinMDR3*/T. The following two DNA fragments were generated from genomic DNA of T. mentagrophytes 1062Av1401 by PCR: the TmeCYP51B1 fragment contains the 5′ untranslated region (UTR) and wild-type open reading frame (ORF) of *TmeCYP51B*, and the TmeCYP51B2 fragment contains the 5′ UTR and mutated ORF of *TmeCYP51B*, with a point mutation leading to the Gly443Glu substitution. The point mutation leading to the Gly443Glu substitution was introduced into the coding region of *TmeCYP51B* by overlap extension PCR with the primers listed in Table S1. The two obtained fragments were digested with SpeI/ApaI and cloned into SpeI/ApaI double-digested pAg1-*TmeCYP51B*/T, resulting in the generation of pAg1-*wTmeCYP51B*/C and pAg1-*mTmeCYP51B*/C, complementation vectors for the *TmeCYP51B* disruptant (Fig. 2C, Table 2).

PCR was performed using PrimeSTAR HS or PrimeSTAR GXL DNA polymerases (TaKaRa Bio). All the internal SpeI, ApaI, BamHI, and KpnI sites contained in the amplified fragments were inactivated by overlap extension PCR with the corresponding pairs of the primers, respectively. Except for the primers used for amplification of the *Ptef1α* promoter, all primers used for amplification of the specific DNA fragments from *T. indotineae* were designed based on the whole-genome sequence of T. mentagrophytes (formerly *A. vanbreuseghemii*) TIMM2789 (47). Nucleotide sequences of the primers used are listed in Table S1. Where necessary, the amplified fragments were gel purified with a QIAEX II gel extraction kit (Qiagen), subcloned into HincII-digested pUC118, and sequenced.

### Fungal genetic transformation.

Plasmid DNAs of pAg1-*TinCYP51B*/OE, pSilent1-*TinCYP51B*, pAg1-*TinCYP51B*/T, and pAg1-*TinMDR3*/T were used as templates for PCR, to amplify the sequences indicated in Fig. 3 and 5. The primer pairs used for PCR were as follows: P60-P61 for pAg1-*TinCYP51B*/OE, T7-M13rv for pSilent1-*TinCYP51B*, P35-P42 for pAg1-*TinCYP51B*/T, and P43-P52 for pAg1-*TinMDR3*/T. The amplified DNA fragments were purified using the QIAquick PCR purification kit (Qiagen), concentrated by ethanol precipitation, and then introduced into each host cell by the protoplast/PEG method, as described previously, with several minor modifications (48). After the PEG treatment, protoplasts were inoculated onto SDA supplemented with 1.2 M d-sorbitol and 1.0% (wt/vol) yeast extract containing 250 μg/mL G418 or hygromycin B (Wako Pure Chemical), and colonies grown on the selective agar plates were isolated for further investigation.

The ATMT system was used for disruption of the *TmeCYP51B* locus in T. mentagrophytes 1062Av1401 by pAg1-*TinCYP51B*/T and complementation of the *TmeCYP51B* disruptant by pAg1-*wTmeCYP51B*/C and pAg1-*mTmeCYP51B*/C, as described previously, with several minor modifications (44). After cocultivation, nylon membranes were transferred onto SDA containing 250 μg/mL G418 or hygromycin B and 200 μg/mL cefotaxime sodium (Sanofi), overlaid with 10 mL SDA supplemented with the same concentration of G418 or hygromycin B and cefotaxime sodium, and incubated at 28°C. After 48 h, the plates were further overlaid with 10 mL SDA containing 400 μg/mL G418 or hygromycin B and 200 μg/mL cefotaxime sodium and then incubated at 25 to 28°C for 4 to 7 days, according to the development of colonies on the surface of the plates. The colonies regenerating on the selective medium were considered putative G418-resistant or hygromycin B-resistant clones and were transferred onto 1/10 SDA supplemented with 100 μg/mL G418 or hygromycin B, 500 μg/mL cycloheximide, and 50 μg/mL chloramphenicol (if necessary).

The desired transformants were finally screened by PCR, Southern blotting, and nucleotide sequencing. Aliquots of 50 to 100 ng of the genomic DNA were used as templates for PCR. For Southern blotting analyses, aliquots of approximately 10 μg of the genomic DNA were digested with an appropriate restriction enzyme, separated by electrophoresis on 0.8% (wt/vol) agarose gels, and transferred onto Hybond-N^+^ membranes (Cytiva). Southern hybridization was performed using an ECL direct nucleic acid labeling and detection system (Cytiva) according to the manufacturer’s instructions.

### Data availability.

The updated sequences were submitted to GenBank with the following identification numbers: OK539857 (*TinCYP51A*), OK500342 (*TinCYP51B* locus of TIMM20114), OM158720 (*TinCYP51B* locus of TIMM20115), OK500343 (*TinCYP51B* locus of TIMM20116), OM158721 (*TinCYP51B* locus of TIMM20117), OK500344 (*TinCYP51B* locus of TIMM20118), OM158722 (*TinCYP51B* locus of TIMM20119), OK539858 (*TinMDR3*), OK513035 (*TinTef1α*), and OK539856 (*TmeCYP51B*). The whole-genome sequences of six *T. indotineae* strains (TIMM20114, TIMM20115, TIMM20116, TIMM20117, TIMM20118, and TIMM20119) were also deposited in GenBank with the following accession numbers: JAJVHL000000000, JAJVHI000000000, JAJVHK000000000, JAJVHH000000000, JAJVHJ000000000, and JAJVHG000000000. The details are shown in Table S2.
