# Supplementary material for: Gene Amplification of CYP51B: a New Mechanism of Resistance to Azole Compounds in Trichophyton indotineae
Source: Antimicrob Agents Chemother. 2022 May 12;66(6):e00059-22. doi: 10.1128/aac.00059-22 (PMC9211412; doi:10.1128/aac.00059-22)
Supplement: Supplemental file 1 — Fig. S1, Tables S1 to S3, and sequences of the TIMM20114 and TIMM20118 CYP51B loci. Download aac.00059-22-s0001.pdf, PDF file, 1.9 MB [file aac.00059-22-s0001.pdf]

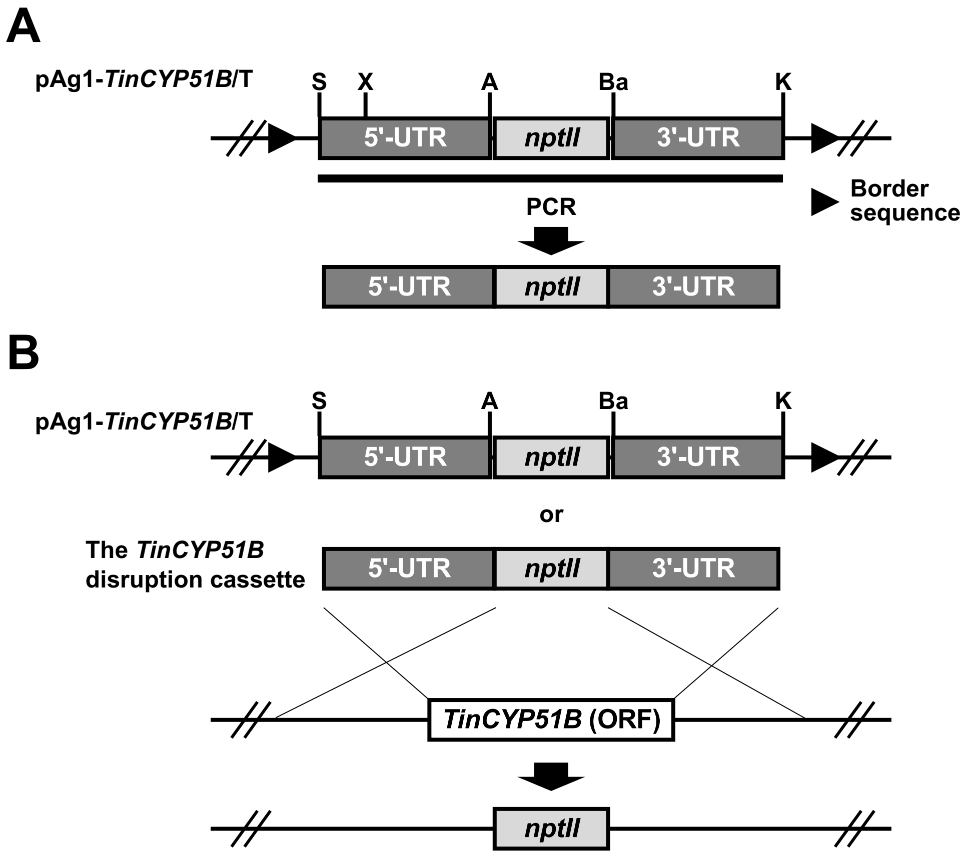

FIG. S1 Disruption of the *TinCYP51B* gene of *T. indotineae* strains with low azole susceptibility by gene replacement strategy. (A) Schematic representation of a part of the *TinCYP51B*-targeting vector pAg1-*TinCYP51B*/T. Plasmid DNA of pAg1-*TinCYP51B*/T was used as a template for PCR, to amplify the sequence indicated by the thick line. The *nptII* cassette is composed of *PtrpC*, *nptII*, and *TcgrA*. Border sequences are the specific regions that delineate the DNA to be transferred during *Agrobacterium tumefaciens*-mediated transformation. Restriction enzyme site: A, ApaI; Ba, BamHI; E, EcoRI; K, KpnI; P, PstI; S, SpeI. (B) Schematic representation of the *TinCYP51B* locus before and after homologous recombination.

TABLE S1 Primers used in this study

Primer name	Sequence (5'-3')	Chain	Purpose
P1	ATGGCCGTGTTCACAGTGG	Sense	Amplification of *TinCYP51A*
P2	TCAATGTGACTGAGGACGTCG	Anti-sense	Amplification of *TinCYP51A*
P3	ATGGGCCTCTTAGCCGAC	Sense	Amplification of *TinCYP51B*
P4	TCATTTCTGGGAAGGCTGC	Anti-sense	Amplification of *TinCYP51B*
P5	TGTTGGTGATGAGGCACAGT	Sense	qRT-PCR (*TinACTIN*)
P6	CCATGTCATCCCAGTTTGTG	Anti-sense	qRT-PCR (*TinACTIN*)
P7	GCGTGACCCAGCCAACA	Sense	qRT-PCR (*TinGAPDH*)
P8	TTGGCACCTCCCTTCAAGT	Anti-sense	qRT-PCR (*TinGAPDH*)
P9	CCTAATGCCCTTCCTGGATT	Sense	qRT-PCR (*TinMDR1*)
P10	AAATTGCCAGCTCGTTCTGT	Anti-sense	qRT-PCR (*TinMDR1*)
P11	TGACTCTGAATCCGAAAAGG	Sense	qRT-PCR (*TinMDR2*)
P12	GTCGGTGAGCAACAGCAATA	Anti-sense	qRT-PCR (*TinMDR2*)
P13	TCACTGGTGAAATGCTCGTTGACG	Sense	qRT-PCR (*TinMDR3*)
P14	GGTTGACGGAGCAAGGCACTG	Anti-sense	qRT-PCR (*TinMDR3*)
P15	GAGACGTAGATATTTCGAGTGCC	Sense	qRT-PCR (*TinCYP51A*)
P16	TTGAGCGTACCTCTTCCCCCTG	Anti-sense	qRT-PCR (*TinCYP51A*)
P17	AAGTTCGTCAAGTTCGGCCT	Sense	qRT-PCR (*TinCYP51B*)
P18	GAGAGGCGGTGTAGATGGTG	Anti-sense	qRT-PCR (*TinCYP51B*)
P19	GCTGATGCCAATGCCATGGAG	Sense	qRT-PCR (*TinMFS2*)
P20	TCGGGTTTCGAGGATCGCTTGG	Anti-sense	qRT-PCR (*TinMFS2*)
P21	AAA*GGATCC*CCACTAAGACTCCTTCAAGCTCCCCA	Sense	Amplification of *Ptef1α* for pAg1-*TinCYP51B*/OE
P22	GGCTAAGAGGCCCATGGTGACGGTGTATTTTTGTG	Anti-sense	Amplification of *Ptef1α* for pAg1-*TinCyp51B*/OE
P23	GCAGCCTTCCCAGAAATGACAGCAGAAGAATCTCT	Sense	Amplification of *TcgrA* for pAg1-*TinCYP51B*/OE
P24	AAAA*GGTACC*AGATGATTCATGA	Anti-sense	Amplification of *TcgrA* for pAg1-*TinCYP51B*/OE
P25	AAATACACCGTCACCATGGGCCTCTTAGCCGACAT	Sense	*TinCYP51B* cDNA

P26	AGAGATTCTTCTGCTGTCATTTCTGGGAAGGCTGC	Anti-sense	*TinCYP51B* cDNA
P27	TTT*CTCGAG*CCTCACCGATGGGAGAACAACGTCGG	Sense	Amplification of the *TinCYP51B* cDNA fragment for pSilent1-*TinCYP51B*; Amplification of the *TinCYP51B*-specific hybridization probe
P28	TTT*AAGCTT*TGGCGGCACAACATCTTTGGTGCCTT	Anti-sense	Amplification of the *TinCYP51B* cDNA fragment for pSilent1-*TinCYP51B*; Amplification of the *TinCYP51B*-specific hybridization probe
P29	TTT*AGATCT*TGGCGGCACAACATCTTTGGTGCCTT	Sense	Amplification of the anti-sense *TinCYP51B* cDNA fragment for pSilent1-*TinCYP51B*
P30	TTT*GGTACC*CCTCACCGATGGGAGAACAACGTCGG	Anti-sense	Amplification of the anti-sense *TinCYP51B* cDNA fragment for pSilent1-*TinCYP51B*
P31	TTT*ACTAGT*CCACTAAGACTCCTTCAAGCT	Sense	Amplification of *Ptef1α* for pSilent1-*TinCYP51B*
P32	TTCTACAGATGGTAACTgGTCGAGAGCAGAGAGTA	Anti-sense	Inactivation of the internal SpeI site by overlap extension PCR
P33	TACTCTCTGCTCTCGACcAGTTACCATCTGTAGAA	Sense	Inactivation of the internal SpeI site by overlap extension PCR
P34	TTT*CTCGAG*GGTGACGGTGTATTTTTGTGTGGTT	Anti-sense	Amplification of *Ptef1α* for pSilent1-*TinCYP51B*
P35	CATG*ACTAGT*GCGGTATGCCTTTACCCATTGCATA	Sense	Amplification of the 5' UTR of *TinCYP51B* gene
P36	AAA*GGGCCC*ATGGTGATCGTCTTATCCTCCTGCT	Anti-sense	Amplification of the 5' UTR of *TinCYP51B* gene; Amplification of the *TmeCYP51B*-specific hybridization probe
P37	CCAG*GGATCC*TAATCACGACTGCGGATTGAATCAT	Sense	Amplification of the 3' UTR of *TinCYP51B* gene
P38	TTATCAGCCATGCGTACcGTACCTTCATCAATCTC	Anti-sense	Inactivation of the internal KpnI site by overlap extension PCR
P39	GAGATTGATGAAGGTACgGTACGCATGGCTGATAA	Sense	Inactivation of the internal KpnI site by overlap extension PCR
P40	CCCTTACCGGTTTACCTtGGCCCTTGGTGCCGTGG	Anti-sense	Inactivation of the internal ApaI site by overlap extension PCR
P41	CCACGGCACCAAGGGCCaAGGTAAACCGGTAAGGG	Sense	Inactivation of the internal ApaI site by overlap extension PCR
P42	CCCA*GGTACC*ATATGTCGACTGCACCGGAATATTG	Anti-sense	Amplification of the 3' UTR of *TinCYP51B* gene
P43	GTGT*ACTagt*ACCGTGGTCATCGTGTAGATGATAT	Sense	Amplification of the 5' UTR of *TinMDR3* gene
P44	TATTGTGAGCCATAGGGaACCGATTTACTCTGAAA	Anti-sense	Inactivation of the internal KpnI site by overlap extension PCR
P45	TTTCAGAGTAAATCGGTtCCCTATGGCTCACAATA	Sense	Inactivation of the internal KpnI site by overlap extension PCR

P46	CAGCGTATGTTCAGGGCCtAATCATATGCCACAGA	Anti-sense	Inactivation of the internal ApaI site by overlap extension PCR
P47	TCTGTGGCATATGATTaGGCCCTGAACATACGCTG	Sense	Inactivation of the internal ApaI site by overlap extension PCR; Amplification of the *TinMDR3*-specific hybridization probe
P48	TTT*<u>GGGCCC</u>*CTGCTGATCTCTGTGGACGATAGCAC	Anti-sense	Amplification of the 5' UTR of *TinMDR3* gene; Amplification of the *TinMDR3*-specific hybridization probe
P49	TTCC*<u>GGATCC</u>*CATGAGCCATTACACATGGGTTACA	Sense	Amplification of the 3' UTR of *TinMDR3* gene
P50	TACCATGGACATGGTACtTGGTGCAACCAGGACGA	Anti-sense	Inactivation of the internal KpnI site by overlap extension PCR
P51	TCGTCCTGGTTGCACCAaGTACCATGTCCATGGTA	Sense	Inactivation of the internal KpnI site by overlap extension PCR
P52	TGA*<u>GGTACC</u>*TACTACTCTGTGAAATCAGTTAACTT	Anti-sense	Amplification of the 3' UTR of *TinMDR3* gene
P53	GGC*<u>ACTAGT</u>*TATGGCTAACCGTTGTCGTAGCATGA	Sense	Amplification of the TmeCYP51B1 and TmeCYP51B2 fragments
P54	AAA*<u>GGGCCC</u>*TCATTTCTGGGAAGGCTGCTTTCGCT	Anti-sense	Amplification of the TmeCYP51B1 and TmeCYP51B2 fragments
P55	AGACAGATTACGaATACGGCTTGGTT	Sense	Generation of a point mutation in *TmeCYP51B*
P56	AACCAAGCCGTATtCGTAATCTGTCT	Anti-sense	Generation of a point mutation in *TmeCYP51B*
P57	TTGACGGACCGTTGATAGCTTAACCTGCCATGACT	Sense	Amplification of the *TmeCYP51B*-specific hybridization probe
P58	TTT*<u>CTCGAG</u>*ATGGGCCTCTTAGCCGACATTGTCTC	Sense	Amplification of the *TinCYP51B*-specific hybridization probe
P59	TTT*<u>AAGCTT</u>*CTTGCAGTCGTCAAAGAACTTGTACG	Anti-sense	Amplification of the *TinCYP51B*-specific hybridization probe
P60	TTC*<u>AGATCT</u>*AGAAGATGATATTGAAGG	Sense	Amplification of the *nptII*/*TinCYP51B* cDNA cassette
P61	CGATTAAGTTGGGTAACGCCAG	Anti-sense	Amplification of the *nptII*/*TinCYP51B* cDNA cassette
P62	ATGAAAAAGCCTGAACTCAC	Sense	Amplification of the *hph* gene
P63	GTTTCCACTATCGGCGAGTA	Anti-sense	Amplification of the *hph* gene
T7	TAATACGACTCACTATAGGG	Sense	Amplification of *TinCYP51B* RNAi cassette
M13rv	CAGGAAACAGCTATGAC	Anti-sense	Amplification of *TinCYP51B* RNAi cassette

Restriction sites are italicized and underlined, and nucleotide substituitions are shown in lower case letters.

TABLE S2 Overall genetic features of six *T. indotineae* strains

Species and isolate no.[a]	BioProject number	GenBank accession number (wgs)	Total Number of scaffolds	N50 (bp)	Longest sequence length (bp)	Total sequence length (bp)	Depth of coverage (×)	GC content (%)
T. indotineae								
TIMM20114 (UKJ1676/17; IFM 67092)	PRJNA780377	JAJVHL000000000	3	8,011,651	10,184,091	22,261,634	40	49
TIMM20115 (UKJ1700/17II; IFM 67093)	PRJNA780377	JAJVHI000000000	6	4,361,119	6,825,914	22,331,438	38	49
TIMM20116 (UKJ1708/17; IFM 67094)	PRJNA780377	JAJVHK000000000	8	4,066,087	7,369,201	22,383,831	46	49
TIMM20117 (200087/18; IFM 67095)	PRJNA780377	JAJVHH000000000	8	3,672,843	6,160,204	22,303,869	32	49
TIMM20118 (UKJ1687/17; IFM 67096)	PRJNA780377	JAJVHJ000000000	6	4,204,240	4,528,007	22,316,814	43	49
TIMM20119 (200123/18; IFM 67097)	PRJNA780377	JAJVHG000000000	8	3,646,268	4,540,326	22,297,103	32	49

[a]All the *T. indotineae* isolates were collected in India and preserved in the culture collection of Teikyo University Institute of Medical Mycology (TIMM) and Medical Mycology Research Center, Chiba University (IFM), through the National Bio-Resource Project, Japan (http://www.nbrp.jp/).

TABLE S3 Conserved sequences HR1 and HR2 that could allow amplification of *CYP51B* in selected dermatophyte species of *Trichophyton* (T.), *Nannizia* (N.) and *Microsporum* (M).

Species	HR1 sequence[a]	HR2 sequence[b]
T. indotineae	**5'-AACCGAAAAGCAGC-3'**	**5'-AAGCGAAAGCAGC-3'**
T. tonsurans	5'AACCGAAAAGCAGC-3'	5'-AAACGAAAGCAGC-3'
T. equinum	5'-AACTGAAAAGCAGC-3'	5'-AAGCGAAAGCAGC-3'
T. interdigitale	5'-AACCAAAAAGCAGC-3'	5'-AAGCGAAAGCAGC-3'
T. rubrum	5'-AACCAAAAAGCAAC-3'	5'-AAACGAAAGCAGG-3'
T.violaceum	5'-AACCAAAAAGCAAC-3'	5'-AAACGAAAGCAGG-3'
T. soudanense	5'-AACCAAAAAGCAAC-3'	5'-AAACGAAAGCAGG-3'
T. verrucosum	5'-AACCGAAAAGCAAA-3'	5'-AAGCGAAAGCAGC-3'
T. benhamiae	5'-AAACGAAAAGCAAA-3'	5'-AAACGAAAGCAGC-3'
N. gypsea	5'-AACCGAAAAGCAGC-3'	5'-AAACGAAAGCAGC-3'
M. canis [c]	-	5'-AAACGAAAGCAGC-3'

[a]The HR1 sequence is centered at about position -640 in relation to the initiator codon of the *CYP51B* gene.

[b]The HR2 sequence is localized within the CYP51B coding region, -27 to -14 nucleotides in relation to the last nucleotide of the stop codon of the *CYP51B* gene.

[c]The sequence in *M. canis* differs too much to identify an HR1-related sequence.

<u>CYP51B locus in TIMM20114</u>

The original 3’ of the *CYP51* gene (in TIMM20118_CYP51B1) is marked in red (corresponding to H109_04528 in *Trichophyton interdigitale* strain MR816).

CYP51B promoter is in violet
CYP51B ORF is in green

The two nearly identical sequences 5′-AACCGAAAAGCAGC-3′ (HR1, for homologous recombination site 1) and 5′-AAGCGAAAGCAGC-3′ (HR2) at the borders of the CYP51B origin block are highlighted.

At 5’.: CAMK protein kinase (corresponding to H109_04528 in *Trichophyton interdigitale* strain MR816)
At 3’: Protein FYV4, mitochondrial (corresponding to H109_04526 in *Trichophyton interdigitale* strain MR816)

The *TinCyp51B* gene in the genome of TIMM20114 (5' to 3')

CATGCAGAGTGCGGTATGCCTTTACCCATTGCATACTCTCTGCCGTCTAGTGATCAATTGCTAA
CCATCTGGGTACTGCAGATTTCAGGATCAAGCTCCCCACGATATCCAACCGCCATTGCCTGATA
TTCCACGAAAACAGGGGAGGAGACTTTGTTGCAATTGTGGAAGATCTCTCTATCAATGGCACTT
TCATCAACGACGCCATCATCGGCAAGAACAAACGCCGAGAGCTCGAAAACGGCGACGAGATTAC
CATCCTGCAAGAATCCAGATTCATCTTCAACTACCCGCGGAGTCGGAACACAAGCAAGTTCCGA
CAGCAGTATAAGCTCTTTGATATGCTGGGTAGGGGACACTTTGCCTCGGTTTATCTATGCGCTG
AACGTTCTACGGGGATCAAGTATGCCGTCAAACATTTCGAGAAACGCCCGGGCGCAGTTCAGAG
GCTAGACCGTGAAGCACTACAGCAAGAGATTTCAATGCTGATGAGTGTCAACCATCCGAACATG
CTGTGCCTAAAGGATACCTTTGACGAGAGCGATGGTGTCTTCCTGATTCTTGAGCTTGCGCCAG
AGGGCGAACTGTTCAACTGGATCATTAGGCATCAGAAACTCAGCGAGGAGGAGACTCGAAAAGT
ATTCATTCAGCTTTTTGAAGGTTTGAGATACCTGGTATGTCGTTCGTTTTCTGGTTGATCAGAC
TCATGGCAGTGTATATGGCTAACCGTTGTCGTAGCATGAACGAAACATCATTCACCGTGACATA
AAGCCAGAGAACATTCTGGTTGTCGACAAAGATCTTACCGTAAAATTAGCAGACTTCGGCCTTG
CAAAGATTGTCGGCGAACACTCCTTTACCACCACGCTGTAAGTTCACCTACCTTCTGCCGTTCA
AATCTGGGGTCAATCTTACTAAGAGATCATAAAATCTAGATGTGGAACGCCTGGCTGTAAGTTT
CCCTGCTGCTCTGTTAACGCTGGTGCTTTCATCCACCAAATGTGAATCTAACAATTCTTATCAG
ATGTTGCTCCTGAGATACTGGCAGAAAACGCTGAAGCCAGAATGTACACCCGAGCGGTTGACAT
CTGGTCACTCGGAGTGGTACTCTACATCTGCCTGTGCGGATTCCCTCCTTTCTCAGATGAACTG
TACCACCCAAGAGACTACCCATACACTCAGAAAGAGCAGATCCAGCTCGGAATATTTAAGTATC
CTCTTCCATACTGGGACTCAATCGATTATAGAGCCATGGACCTTATCGACTCCATGATCGAAGT
CGACGTGAAGGAGCGTTTGAAAGTAGATGAGTGTCTCCAGCACCCATGGATCACTGGCATCGAC
CCCGATGAGCCTAGGGTGGCAGACAGCACCGATGACCTAGCCGGGGCCATCGGTGGCCTGAGGG
TGTCCAAAGAACGCCGGCCAACTATGGTCCGCAGGCTGTTGAGTGACATACATGAAGTCACGGT
CAAGCAGGTGGTGAAGACCAAGGAAGACGACCCTGCTTCCCAGGACATAACTATATACCAGAAT
CATCCTGTGCCTCGAGCCCAGGAGCCCAAACCGGCTGGCAACCGGAACTCAAAGGAGTTCATGG
AACTCGGTGGAAGTGGAGACCAGGTGTTATTCGATGAGCAGTCTGACGACTCGCTGGAGCTTGA
GAGTGAGGCACGGAAGCCAGTTGTCCACCAAGACGACGCAGACAAGACTCCACGAGAAAGAAAG
AGGTAGCAGAAGCCCGATCCGTCTTGGGATGGTATTCTGCTAGCTATACCGTGCTATTCCGGAC
TTTTGTTTCCAATTCCTTTTTCGTTTATATTGATTGTGATCTTTCTTAATTTTTTCTTTAACTA
TACCCCCCGGTTGAAAGGTGATAGGGGGCCAACTAACCGAAAAGCAGCAGAAAAACCGGCTTGG
TCGCTGCCTTGGAGACGACGGCGATTCTCGTGTTTAGGATTTCATTTTTGAGTGATATAACTAT

GGGTACAATAGCGTCCACTGGGCAATGTAATTACAGTCTCTTCTCACTCACCACCGGACAATAA
TAACAATATTAATATTGACGGACCGTTGATAGCTTAACCTGCCATGACTAAGCCGGCCTCCCTG
AAGCCGCGATGGCCTGATGATGCGCCCACCGTCAGGTCTCGCTCGCTCAGGCAGCAGAGAATCC
AGCGGAGGAGATGACGCCCTGCACGAACACCGTCCAGGGACCGTCTCCGCCCCGTCGCGAGCTC
GAATTGTAATCATCTCGACGGCCGTTGGCCTGCTCGCCGCAAGGGAGAAAAAGGGGGGCAGAAG
GGCAGCGGTAATTTTGGTATTGGAGATGCAGCTGTAAACCAATAAAAAACGACGTAAAATACCA
GGAACAATGCATTATCTTACAGTACCGTACGCCCCTTGCATAAAATGCGAGTTCGATCTCAAGA
TGCAACTTTTTTTTTCCCTTGCTTCGTCCGCAGCAGCTTCAAGGCATTCATCACAAACAACGAA
CAAGTCTTGAGCGGAAGCAGGAGGATAAGACGATCACC**ATG**GGCCTCTTAGCCGACATTGTCTC
TCGTTTCTGCGAGAACTGCTCGACCCTGTCCACCGCCGCGCTCGTCGCAAGTGCCGTATCGGCT
TTTATCGTCCTCTCCATTGTTATCAACGTCCTGCAGCAGCTCCTGTTCAAGGACCCTACAAAGC
CTCCGGTGGTCTTCCACTGGGTTCCGGTCATTGGAAGCACGATCTCCTATGGAATTGACCCGTA
CAAGTTCTTTGACGACTGCAAGGAGAAGGTTAGTTGGTGCAATTCAACTTAGCTATATACCAGG
TTGCGCAAAGCTAACGTATAAATAATTTCGGTACTCTAGTATGGAGACATCTTCACATTCATAC
TGCTGGGCAAGAAGACGACTGTTTTTCTCGGTACAAAGGGAAATGATTTCATTTTGAACGGCAA
GCTCAAGGATGTTTGCGCGGAGGATGTCTACTCCCCCCTCACCACCCCAGTGTTCGGACGACAT
GTGGTGTATGATTGCCCAAACTCCAAGCTCATGGAGCAGAAGAAGGTGTGTACAGCAATTTATT
CCCGTCTACATCTCCTGGGCCTCCATTGCTAATTTAATCGTCTACCAGTTCGTCAAGTTCGGCC
TCACCTCTGAAGCTCTCCGATCCTATGTCACCCTGATCACCAAGGAAGTTGAGCAGTTCTTCGA
GTCCTCCCCCGTCTTCAAGGGCGACTCCGGAGTTTTCAACGTCAGCAAGGTCATGGCTGAAATC
ACCATCTACACCGCCTCTCGATCTCTACAGGGCAAGGAGGTGCGAGGAAAGTTCGATTCCAGCT
TTGCGGAACTCTACTCCGATCTCGACATGGGCTTCGCCGCCATCAACTTCATGTTCCCATGGTT
CCCCTTCCCACACAACCGCAAGCGTGACCGTGCTCAAAAGAAGATGGCCCAGGTTTACACCGAC
ATCATCCGTCAGCGACGTGAGGCTGGTGGAGAGAAAGACTCCGAGGACATGGTATGGAACTTGA
TGTCGTCCGTGTACAAGAATGGAACGCCAATTCCAGATATCGAAGTCGCCCACATGATGATTGC
TCTTCTTATGGCTGGCCAGCACTCTTCTTCCTCCACCGGCTCCTGGATCGTTCTCCGCCTTGCC
AGCCGTCCAGATATTCTCGAGGAACTCTACGAGGAACAGAAACGTGTTCTCGGCGAGGATCTTC
CACCACTCACCTACGAATCTCTCCAGAAACTTGATCTTCACAACAATGTAATCAAGGAGACTCT
CCGCCTCCACGCTCCCATCCACTCTATCCTCCGTGCTGTTAAATCCCCTATGCCCGTTGAAGGA
ACTAACTATGTTGTCCCAACCTCTCACAACCTCCTTGCCGCTCCTGGTGTTCCCTCACGAGACC
CTCAGTACTTCCCTGACCCTCTTGTTTGGAACCCTCACCGATGGGAGAACAACGTCGGTGTCAC
CGTAGTCGAGGCCAGTGAAGAAAAGACAGATTACG**G**ATACGGTTTGGTTAGCAAGGGTGCCAAC
AGCCCTTACCTCCCATTCGGCTCAGGCAGACACAGATGCATCGGTGAACAATTTGCATATGTTC
AGCTTGGAACCGTAACAGCTACGTTAGCCAGACTAATGAAATGGAAGCAAGTTGAAGGCACCAA
AGATGTTGTGCCGCCAACTGACTATTCGGTAAGTTTGGCATATTACGCTCCTTTTTATTTTGCT
CGATTTTACTAACTTTGCATTAATAGTCCCTCTTCTCGAAGCCCCTTGGCGACCCAATGGTCTC
GTGGGAGAAGCGAAAGCAGCCTTCCCAGAAA**TGA**TAATCACGACTGCGGATTGAATCATGTTAA
ATTTCTTGCTTCTTGCTAATTTATTTCTTCTCTTTTCTTACGTTTATACACCGAGCCATATTTG
CACTTTATGGTCACGCAAATAGGTTTTCGGAATAGAGCATATGTAATAAAAACTTTCTTTTGAC
CATGCTTTTGAAGATGGGGGATGTAGATAATAGTATTATTCTAATACAGCCATTTATTACAATA
CTCTAGATGAACAGGAAAGTTGCAGTTACTCCGTTAGCTTTGGTGCAGAAACAAAATGGTAATA
TACATTGGAAATAGTACATCTTCCCGTAGCTGGGATAATCTAGCATCTAAACAGCGATATCCAA
GCCAGTAATGTCAGGAAGTAACCAGCCAGCAACGTCGTTAATCCCGTGCCCCCTTTCAACTCCG
ACGCTACAAAACGCCTAAGCAGCTGCCTGAGCATTCCTCCTCTCCGCCAACGCTCTCTTCGCTC
TGATCTCCTTCTGTCTTCTCTGACCACCGTCAATCTTTCTGCCACGCTTATGCTCCCACATGCC
ATCAGTAACCTGAACCTTGACGGCGGTACTCCATGTGTTTGGAATAATCTGAGCATACGGCCCC
TTGATCATATTCCCGTGGTGCAATTTATAATGGGCAAACTTTTTGAGTGGCTTGGAAGTATCCT
TGAGCTCTGTTTCGCCTGGTGGTAGATTGACAATCGCATAACGCATTCCAGGTGTAAGGGTAGC
GGTGCCCATTGTGCTAACATCTAGGGACTCTCCGTCGGTGGCATTGGTAGTTTTTGCACTCTTT
GACTTTTCGACGGGGACCTCAACAGCTCTCAGCTCAGCAACACCGTCAACAACGTTTTCGAAAT
CACCTCCTGGGCCGTAGTCCTGCTGTCTGAATTTCTCTCTCCACCGCAGTATGTATCGTCTGTC
CCTTGTCATTTCTATGCCCAATTCTCGAAGCTCAGCCGAGTCCGCTGTAAACAATTGCTTCCAT

CCGGTGATTTTGCTGGAATATCTGGACATGTTTCGCCCAATTAGGCGTAGGAATGTTTCGACAT
CGGGCACGAATGGGGTAGGTGATGGCACTGTTGCGGGTTTTCGAGATTGATGAAGGTACCGTAC
GCATGGCTGATAATGGGGGTTTGTGACGGAAGTGAAGAGCCGTAGTGGAAGGGATGTTCTGGCA
TTAAAGCCCGGTATGCGCGCCGCAGCCATAGTGGTTATATCGCCTTATATCAAACCGTTCAATG
TCGAGATCTGGGGGGTGAAGATTTTAATTTAGCTCGTGGTGTAAGAACACCATCGGCGGGCGTG
ACGAAAGCGAAAAAAGCCACGGCACCAAGGGCCCAGGTAAACCGGTAAGGGACTCAAGGCGAGT
CAATCCAATGCTCAATGGCGCTGTATAGAAGATGTTCAACTGGTTCTTCGACTTCGTTCAAGAT
TTTGCACCAAAATTTTGAATTAGGTCCGACGGCCTTAGCTCGGATCAACAAAACGCCGATCTCC
GGTGAGCTCGGGCCAGCCGAAGCTTCCATCGCAACATTCAAAGATGAAAGACGACGATATATAT
TTTGATAACAAACAAAGCTGTATTGTTGATTGAATACTAGGAGAGCTGCTCAGAGCAGTGTCTC
ACTCTTGTGCCACTCTATCCTTTGTCTCGCGAAATTGGTGGAAGCCAATTTCGCTTGACATTAT
GGCAGTGTCGCCGCTTCCCTCTCAATTCCCTCCCATCACTACTACCGGTGCTCCAAAGTCGTTA
GCGATGAGAAATATTGCTTCTCAAGAGCCTCGGACGGCGCCTAGTGGTGATCCTGCAGTTCCTA
AGCCCTCCATGCCCGAACCAGAGCTGGCGGAGCAGTTAAACGCTGATGTACGGCGGAAATATGT
CAAAGGTGGGCGGGCAAATACACCCCAACCATTGGGACCCCATAATGCTAACATTTGGTTATTC
TGAAAGATAAGAAGCTCGGTGAAGGTACATATGCAGTTGTTTACCTAGGCCATCTACGAGATGA
TCCCACTTCGCTCGTTGCAATCAAGAAAATAAAACTAAACGCCGAGTACAAGGACGGGCTATCC
ATGGACGCCATCCGTGAAGTAAAGTACCTTCAGGAACTGTCTCACCCGAACGTAATTGCGTTGC
ATGACGTTTTCTCCTCAAAGGATCAAAATCTAAACCTCGTCCTTGAATTTTTACCCCTTGGAGA
TCTTGAAATGTTGATCAAAGACAACTCTATCCAGTACGGTGTGGCTGATATAAAAGCATGGATA
AGCATGCTTGCACGTGGAGTGTGGTTCTGTCATAAGAATTTCATTCTACACCGTGATATTAAGC
CTAACAACTTATTAATTGCATCGGATGGAGAAGTTAAATTAGCGGATTTTGGTCTTGCCAGATC
CTTTGCCGATCCATATCTCAACATGACACATCAAGTCATCACAAGATGGTACAGGCCATTAGAA
CTACTCTTCGGCGCCAGGCAATATTCCGGTGCAGTCGACATATGGTCTATGGG

CYP51B locus in TIMM20118

The original 3’ of the *CYP51* gene (in TIMM20118_CYP51B1) is marked in red (corresponding to H109_04528 in *Trichophyton interdigitale* strain MR816).

CYP51B promoters are in violet
CYP51B alleles are in green

The two nearly identical sequences 5′-AACCGAAAAGCAGC-3′ (HR1, for homologous recombination site 1) and 5′-AAGCGAAAGCAGC-3′ (HR2) at the borders of the CYP51B origin block are highlighted.

At 5’.: CAMK protein kinase (corresponding to H109_04528 in *Trichophyton interdigitale* strain MR816).

At 3’: Protein FYV4, mitochondrial (corresponding to H109_04526 in *Trichophyton interdigitale* strain MR816).

The 7 *TinCyp51B* gene tandem repeats in the genome of TIMM20118 (5' to 3')

```
CATGCAGAGTGCGGTATGCCTTTACCCATTGCATACTCTCTGCCGTCTAGTGATCAATTGCTAA
CCATCTGGGTACTGCAGATTTCAGGATCAAGCTCCCCACGATATCCAACCGCCATTGCCTGATA
TTCCACGAAAACAGGGGAGGAGACTTTGTTGCAATTGTGGAAGATCTCTCTATCAATGGCACTT
TCATCAACGACGCCATCATCGGCAAGAACAAACGCCGAGAGCTCGAAAACGGCGACGAGATTAC
CATCCTGCAAGAATCCAGATTCATCTTCAACTACCCGCGGAGTCGGAACACAAGCAAGTTCCGA
CAGCAGTATAAGCTCTTTGATATGCTGGGTAGGGGACACTTTGCCTCGGTTTATCTATGCGCTG
AACGTTCTACGGGGATCAAGTATGCCGTCAAACATTTCGAGAAACGCCCGGGCGCAGTTCAGAG
GCTAGACCGTGAAGCACTACAGCAAGAGATTTCAATGCTGATGAGTGTCAACCATCCGAACATG
CTGTGCCTAAAGGATACCTTTGACGAGAGCGATGGTGTCTTCCTGATTCTTGAGCTTGCGCCAG
AGGGCGAACTGTTCAACTGGATCATTAGGCATCAGAAACTCAGCGAGGAGGAGACTCGAAAAGT
ATTCATTCAGCTTTTTGAAGGTTTGAGATACCTGGTATGTCGTTCGTTTTCTGGTTGATCAGAC
TCATGGCAGTGTATATGGCTAACCGTTGTCGTAGCATGAACGAAACATCATTCACCGTGACATA
AAGCCAGAGAACATTCTGGTTGTCGACAAAGATCTTACCGTAAAATTAGCAGACTTCGGCCTTG
CAAAGATTGTCGGCGAACACTCCTTTACCACCACGCTGTAAGTTCACCTACCTTCTGCCGTTCA
AATCTGGGGTCAATCTTACTAAGAGATCATAAAATCTAGATGTGGAACGCCTGGCTGTAAGTTT
CCCTGCTGCTCTGTTAACGCTGGTGCTTTCATCCACCAAATGTGAATCTAACAATTCTTATCAG
ATGTTGCTCCTGAGATACTGGCAGAAAACGCTGAAGCCAGAATGTACACCCGAGCGGTTGACAT
CTGGTCACTCGGAGTGGTACTCTACATCTGCCTGTGCGGATTCCCTCCTTTCTCAGATGAACTG
TACCACCCAAGAGACTACCCATACACTCAGAAAGAGCAGATCCAGCTCGGAATATTTAAGTATC
CTCTTCCATACTGGGACTCAATCGATTATAGAGCCATGGACCTTATCGACTCCATGATCGAAGT
CGACGTGAAGGAGCGTTTGAAAGTAGATGAGTGTCTCCAGCACCCATGGATCACTGGCATCGAC
CCCGATGAGCCTAGGGTGGCAGACAGCACCGATGACCTAGCCGGGGCCATCGGTGGCCTGAGGG
TGTCCAAAGAACGCCGGCCAACTATGGTCCGCAGGCTGTTGAGTGACATACATGAAGTCACGGT
CAAGCAGGTGGTGAAGACCAAGGAAGACGACCCTGCTTCCCAGGACATAACTATATACCAGAAT
CATCCTGTGCCTCGAGCCCAGGAGCCCAAACCGGCTGGCAACCGGAACTCAAAGGAGTTCATGG
AACTCGGTGGAAGTGGAGACCAGGTGTTATTCGATGAGCAGTCTGACGACTCGCTGGAGCTTGA
GAGTGAGGCACGGAAGCCAGTTGTCCACCAAGACGACGCAGACAAGACTCCACGAGAAAGAAAG
AGGTAGCAGAAGCCCGATCCGTCTTGGGATGGTATTCTGCTAGCTATACCGTGCTATTCCGGAC
TTTTGTTTCCAATTCCTTTTTCGTTTATATTGATTGTGATCTTTCTTAATTTTTTCTTTAACTA
```

TACCCCCCGGTTGAAAGGTGATAGGGGGCCAACTAACCGAAAAGCAGCAGAAAAACCGGCTTGG
TCGCTGCCTTGGAGACGACGGCGATTCTCGTGTTTAGGATTTCATTTTTGAGTGATATAACTAT
GGGTACAATAGCGTCCACTGGGCAATGTAATTACAGTCTCTTCTCACTCACCACCGGACAATAA
TAACAATATTAATATTGACGGACCGTTGATAGCTTAACCTGCCATGACTAAGCCGGCCTCCCTG
AAGCCGCGATGGCCTGATGATGCGCCCACCGTCAGGTCTCGCTCGCTCAGGCAGCAGAGAATCC
AGCGGAGGAGATGACGCCCTGCACGAACACCGTCCAGGGACCGTCTCCGCCCCGTCGCGAGCTC
GAATTGTAATCATCTCGACGGCCGTTGGCCTGCTCGCCGCAAGGGAGAAAAAGGGGGGCAGAAG
GGCAGCGGTAATTTTGGTATTGGAGATGCAGCTGTAAACCAATAAAAAACGACGTAAAATACCA
GGAACAATGCATTATCTTACAGTACCGTACGCCCCTTGCATAAAATGCGAGTTCGATCTCAAGA
TGCAACTTTTTTTTTCCCTTGCTTCGTCCGCAGCAGCTTCAAGGCATTCATCACAAACAACGAA
CAAGTCTTGAGCGGAAGCAGGAGGATAAGACGATCACC**ATG**GGCCTCTTAGCCGACATTGTCTC
TCGTTTCTGCGAGAACTGCTCGACCCTGTCCACCGCCGCGCTCGTCGCAAGTGCCGTATCGGCT
TTTATCGTCCTCTCCATTGTTATCAACGTCCTGCAGCAGCTCCTGTTCAAGGACCCTACAAAGC
CTCCGGTGGTCTTCCACTGGGTTCCGGTCATTGGAAGCACGATCTCCTATGGAATTGACCCGTA
CAAGTTCTTTGACGACTGCAAGGAGAAGGTTAGTTGGTGCAATTCAACTTAGCTATATACCAGG
TTGCGCAAAGCTAACGTATAAATAATTTCGGTACTCTAGTATGGAGACATCTTCACATTCATAC
TGCTGGGCAAGAAGACGACTGTTTTTCTCGGTACAAAGGGAAATGATTTCATTTTGAACGGCAA
GCTCAAGGATGTTTGCGCGGAGGATGTCTACTCCCCCCTCACCACCCCAGTGTTCGGACGACAT
GTGGTGTATGATTGCCCAAACTCCAAGCTCATGGAGCAGAAGAAGGTGTGTACAGCAATTTATT
CCCGTCTACATCTCCTGGGCCTCCATTGCTAATTTAATCGTCTACCAGTTCGTCAAGTTCGGCC
TCACCTCTGAAGCTCTCCGATCCTATGTCACCCTGATCACCAAGGAAGTTGAGCAGTTCTTCGA
GTCCTCCCCCGTCTTCAAGGGCGACTCCGGAGTTTTCAACGTCAGCAAGGTCATGGCTGAAATC
ACCATCTACACCGCCTCTCGATCTCTACAGGGCAAGGAGGTGCGAGGAAAGTTCGATTCCAGCT
TTGCGGAACTCTACTCCGATCTCGACATGGGCTTCGCCGCCATCAACTTCATGTTCCCATGGTT
CCCCTTCCCACACAACCGCAAGCGTGACCGTGCTCAAAAGAAGATGGCCCAGGTTTACACCGAC
ATCATCCGTCAGCGACGTGAGGCTGGTGGAGAGAAAGACTCCGAGGACATGGTATGGAACTTGA
TGTCGTCCGTGTACAAGAATGGAACGCCAATTCCAGATATCGAAGTCGCCCACATGATGATTGC
TCTTCTTATGGCTGGCCAGCACTCTTCTTCCTCCACCGGCTCCTGGATCGTTCTCCGCCTTGCC
AGCCGTCCAGATATTCTCGAGGAACTCTACGAGGAACAGAAACGTGTTCTCGGCGAGGATCTTC
CACCACTCACCTACGAATCTCTCCAGAAACTTGATCTTCACAACAATGTAATCAAGGAGACTCT
CCGCCTCCACGCTCCCATCCACTCTATCCTCCGTGCTGTTAAATCCCCTATGCCCGTTGAAGGA
ACTAACTATGTTGTCCCAACCTCTCACAACCTCCTTGCCGCTCCTGGTGTTCCCTCACGAGACC
CTCAGTACTTCCCTGACCCTCTTGTTTGGAACCCTCACCGATGGGAGAACAACGTCGGTGTCAC
CGTAGTCGAGGCCAGTGAAGAAAAGACAGATTACGAATACGGTTTGGTTAGCAAGGGTGCCAAC
AGCCCTTACCTCCCATTCGGCTCAGGCAGACACAGATGCATCGGTGAACAATTTGCATATGTTC
AGCTTGGAACCGTAACAGCTACGTTAGCCAGACTAATGAAATGGAAGCAAGTTGAAGGCACCAA
AGATGTTGTGCCGCCAACTGACTATTCGGTAAGTTTGGCATATTACGCTCCTTTTTATTTTGCT
CGATTTTACTAACTTTGCATTAATAGTCCCTCTTCTCGAAGCCCCTTGGCGACCCAATGGTCTC
GTGGGAGAAGCGAAAGCAGCAGAAAAACCGGCTTGGTCGCTGCCTTGGAGACGACGGCGATTCT
CGTGTT**TAG**GATTTCATTTTTGAGTGATATAACTATGGGTACAATAGCGTCCACTGGGCAATGT
AATTACAGTCTCTTCTCACTCACCACCGGACAATAATAACAATATTAATATTGACGGACCGTTG
ATAGCTTAACCTGCCATGACTAAGCCGGCCTCCCTGAAGCCGCGATGGCCTGATGATGCGCCCA
CCGTCAGGTCTCGCTCGCTCAGGCAGCAGAGAATCCAGCGGAGGAGATGACGCCCTGCACGAAC
ACCGTCCAGGGACCGTCTCCGCCCCGTCGCGAGCTCGAATTGTAATCATCTCGACGGCCGTTGG
CCTGCTCGCCGCAAGGGAGAAAAAGGGGGGCAGAAGGGCAGCGGTAATTTTGGTATTGGAGATG
CAGCTGTAAACCAATAAAAAACGACGTAAAATACCAGGAACAATGCATTATCTTACAGTACCGT
ACGCCCCTTGCATAAAATGCGAGTTCGATCTCAAGATGCAACTTTTTTTTTCCCTTGCTTCGTC
CGCAGCAGCTTCAAGGCATTCATCACAAACAACGAACAAGTCTTGAGCGGAAGCAGGAGGATAA
GACGATCACC**ATG**GGCCTCTTAGCCGACATTGTCTCTCGTTTCTGCGAGAACTGCTCGACCCTG
TCCACCGCCGCGCTCGTCGCAAGTGCCGTATCGGCTTTTATCGTCCTCTCCATTGTTATCAACG
TCCTGCAGCAGCTCCTGTTCAAGGACCCTACAAAGCCTCCGGTGGTCTTCCACTGGGTTCCGGT
CATTGGAAGCACGATCTCCTATGGAATTGACCCGTACAAGTTCTTTGACGACTGCAAGGAGAAG

GTTAGTTGGTGCAATTCAACTTAGCTATATACCAGGTTGCGCAAAGCTAACGTATAAATAATTT
CGGTACTCTAGTATGGAGACATCTTCACATTCATACTGCTGGGCAAGAAGACGACTGTTTTTCT
CGGTACAAAGGGAAATGATTTCATTTTGAACGGCAAGCTCAAGGATGTTTGCGCGGAGGATGTC
TACTCCCCCCTCACCACCCCAGTGTTCGGACGACATGTGGTGTATGATTGCCCAAACTCCAAGC
TCATGGAGCAGAAGAAGGTGTGTACAGCAATTTATTCCCGTCTACATCTCCTGGGCCTCCATTG
CTAATTTAATCGTCTACCAGTTCGTCAAGTTCGGCCTCACCTCTGAAGCTCTCCGATCCTATGT
CACCCTGATCACCAAGGAAGTTGAGCAGTTCTTCGAGTCCTCCCCCGTCTTCAAGGGCGACTCC
GGAGTTTTCAACGTCAGCAAGGTCATGGCTGAAATCACCATCTACACCGCCTCTCGATCTCTAC
AGGGCAAGGAGGTGCGAGGAAAGTTCGATTCCAGCTTTGCGGAACTCTACTCCGATCTCGACAT
GGGCTTCGCCGCCATCAACTTCATGTTCCCATGGTTCCCCTTCCCACACAACCGCAAGCGTGAC
CGTGCTCAAAAGAAGATGGCCCAGGTTTACACCGACATCATCCGTCAGCGACGTGAGGCTGGTG
GAGAGAAAGACTCCGAGGACATGGTATGGAACTTGATGTCGTCCGTGTACAAGAATGGAACGCC
AATTCCAGATATCGAAGTCGCCCACATGATGATTGCTCTTCTTATGGCTGGCCAGCACTCTTCT
TCCTCCACCGGCTCCTGGATCGTTCTCCGCCTTGCCAGCCGTCCAGATATTCTCGAGGAACTCT
ACGAGGAACAGAAACGTGTTCTCGGCGAGGATCTTCCACCACTCACCTACGAATCTCTCCAGAA
ACTTGATCTTCACAACAATGTAATCAAGGAGACTCTCCGCCTCCACGCTCCCATCCACTCTATC
CTCCGTGCTGTTAAATCCCCTATGCCCGTTGAAGGAACTAACTATGTTGTCCCAACCTCTCACA
ACCTCCTTGCCGCTCCTGGTGTTCCCTCACGAGACCCTCAGTACTTCCCTGACCCTCTTGTTTG
GAACCCTCACCGATGGGAGAACAACGTCGGTGTCACCGTAGTCGAGGCCAGTGAAGAAAAGACA
GATTACGAATACGGTTTGGTTAGCAAGGGTGCCAACAGCCCTTACCTCCCATTCGGCTCAGGCA
GACACAGATGCATCGGTGAACAATTTGCATATGTTCAGCTTGGAACCGTAACAGCTACGTTAGC
CAGACTAATGAAATGGAAGCAAGTTGAAGGCACCAAAGATGTTGTGCCGCCAACTGACTATTCG
GTAAGTTTGGCATATTACGCTCCTTTTTATTTTGCTCGATTTTACTAACTTTGCATTAATAGTC
CCTCTTCTCGAAGCCCCTTGGCGACCCAATGGTCTCGTGGGAGAAGCGAAAGCAGCAGAAAAAC
CGGCTTGGTCGCTGCCTTGGAGACGACGGCGATTCTCGTGTT**TAG**GATTTCATTTTTGAGTGAT
ATAACTATGGGTACAATAGCGTCCACTGGGCAATGTAATTACAGTCTCTTCTCACTCACCACCG
GACAATAATAACAATATTAATATTGACGGACCGTTGATAGCTTAACCTGCCATGACTAAGCCGG
CCTCCCTGAAGCCGCGATGGCCTGATGATGCGCCCACCGTCAGGTCTCGCTCGCTCAGGCAGCA
GAGAATCCAGCGGAGGAGATGACGCCCTGCACGAACACCGTCCAGGGACCGTCTCCGCCCCGTC
GCGAGCTCGAATTGTAATCATCTCGACGGCCGTTGGCCTGCTCGCCGCAAGGGAGAAAAAGGGG
GGCAGAAGGGCAGCGGTAATTTTGGTATTGGAGATGCAGCTGTAAACCAATAAAAAACGACGTA
AAATACCAGGAACAATGCATTATCTTACAGTACCGTACGCCCCTTGCATAAAATGCGAGTTCGA
TCTCAAGATGCAACTTTTTTTTTCCCTTGCTTCGTCCGCAGCAGCTTCAAGGCATTCATCACAA
ACAACGAACAAGTCTTGAGCGGAAGCAGGAGGATAAGACGATCACC**ATG**GGCCTCTTAGCCGAC
ATTGTCTCTCGTTTCTGCGAGAACTGCTCGACCCTGTCCACCGCCGCGCTCGTCGCAAGTGCCG
TATCGGCTTTTATCGTCCTCTCCATTGTTATCAACGTCCTGCAGCAGCTCCTGTTCAAGGACCC
TACAAAGCCTCCGGTGGTCTTCCACTGGGTTCCGGTCATTGGAAGCACGATCTCCTATGGAATT
GACCCGTACAAGTTCTTTGACGACTGCAAGGAGAAGGTTAGTTGGTGCAATTCAACTTAGCTAT
ATACCAGGTTGCGCAAAGCTAACGTATAAATAATTTCGGTACTCTAGTATGGAGACATCTTCAC
ATTCATACTGCTGGGCAAGAAGACGACTGTTTTTCTCGGTACAAAGGGAAATGATTTCATTTTG
AACGGCAAGCTCAAGGATGTTTGCGCGGAGGATGTCTACTCCCCCCTCACCACCCCAGTGTTCG
GACGACATGTGGTGTATGATTGCCCAAACTCCAAGCTCATGGAGCAGAAGAAGGTGTGTACAGC
AATTTATTCCCGTCTACATCTCCTGGGCCTCCATTGCTAATTTAATCGTCTACCAGTTCGTCAA
GTTCGGCCTCACCTCTGAAGCTCTCCGATCCTATGTCACCCTGATCACCAAGGAAGTTGAGCAG
TTCTTCGAGTCCTCCCCCGTCTTCAAGGGCGACTCCGGAGTTTTCAACGTCAGCAAGGTCATGG
CTGAAATCACCATCTACACCGCCTCTCGATCTCTACAGGGCAAGGAGGTGCGAGGAAAGTTCGA
TTCCAGCTTTGCGGAACTCTACTCCGATCTCGACATGGGCTTCGCCGCCATCAACTTCATGTTC
CCATGGTTCCCCTTCCCACACAACCGCAAGCGTGACCGTGCTCAAAAGAAGATGGCCCAGGTTT
ACACCGACATCATCCGTCAGCGACGTGAGGCTGGTGGAGAGAAAGACTCCGAGGACATGGTATG
GAACTTGATGTCGTCCGTGTACAAGAATGGAACGCCAATTCCAGATATCGAAGTCGCCCACATG
ATGATTGCTCTTCTTATGGCTGGCCAGCACTCTTCTTCCTCCACCGGCTCCTGGATCGTTCTCC
GCCTTGCCAGCCGTCCAGATATTCTCGAGGAACTCTACGAGGAACAGAAACGTGTTCTCGGCGA

GGATCTTCCACCACTCACCTACGAATCTCTCCAGAAACTTGATCTTCACAACAATGTAATCAAG
GAGACTCTCCGCCTCCACGCTCCCATCCACTCTATCCTCCGTGCTGTTAAATCCCCTATGCCCG
TTGAAGGAACTAACTATGTTGTCCCAACCTCTCACAACCTCCTTGCCGCTCCTGGTGTTCCCTC
ACGAGACCCTCAGTACTTCCCTGACCCTCTTGTTTGGAACCCTCACCGATGGGAGAACAACGTC
GGTGTCACCGTAGTCGAGGCCAGTGAAGAAAAGACAGATTACGAATACGGTTTGGTTAGCAAGG
GTGCCAACAGCCCTTACCTCCCATTCGGCTCAGGCAGACACAGATGCATCGGTGAACAATTTGC
ATATGTTCAGCTTGGAACCGTAACAGCTACGTTAGCCAGACTAATGAAATGGAAGCAAGTTGAA
GGCACCAAAGATGTTGTGCCGCCAACTGACTATTCGGTAAGTTTGGCATATTACGCTCCTTTTT
ATTTTGCTCGATTTTACTAACTTTGCATTAATAGTCCCTCTTCTCGAAGCCCCTTGGCGACCCA
ATGGTCTCGTGGGAGAAGCGAAAGCAGCAGAAAAACCGGCTTGGTCGCTGCCTTGGAGACGACG
GCGATTCTCGTGTT**TAG**GATTTCATTTTTGAGTGATATAACTATGGGTACAATAGCGTCCACTG
GGCAATGTAATTACAGTCTCTTCTCACTCACCACCGGACAATAATAACAATATTAATATTGACG
GACCGTTGATAGCTTAACCTGCCATGACTAAGCCGGCCTCCCTGAAGCCGCGATGGCCTGATGA
TGCGCCCACCGTCAGGTCTCGCTCGCTCAGGCAGCAGAGAATCCAGCGGAGGAGATGACGCCCT
GCACGAACACCGTCCAGGGACCGTCTCCGCCCCGTCGCGAGCTCGAATTGTAATCATCTCGACG
GCCGTTGGCCTGCTCGCCGCAAGGGAGAAAAAGGGGGGCAGAAGGGCAGCGGTAATTTTGGTAT
TGGAGATGCAGCTGTAAACCAATAAAAAACGACGTAAAATACCAGGAACAATGCATTATCTTAC
AGTACCGTACGCCCCTTGCATAAAATGCGAGTTCGATCTCAAGATGCAACTTTTTTTTTCCCTT
GCTTCGTCCGCAGCAGCTTCAAGGCATTCATCACAAACAACGAACAAGTCTTGAGCGGAAGCAG
GAGGATAAGACGATCACC**ATG**GGCCTCTTAGCCGACATTGTCTCTCGTTTCTGCGAGAACTGCT
CGACCCTGTCCACCGCCGCGCTCGTCGCAAGTGCCGTATCGGCTTTTATCGTCCTCTCCATTGT
TATCAACGTCCTGCAGCAGCTCCTGTTCAAGGACCCTACAAAGCCTCCGGTGGTCTTCCACTGG
GTTCCGGTCATTGGAAGCACGATCTCCTATGGAATTGACCCGTACAAGTTCTTTGACGACTGCA
AGGAGAAGGTTAGTTGGTGCAATTCAACTTAGCTATATACCAGGTTGCGCAAAGCTAACGTATA
AATAATTTCGGTACTCTAGTATGGAGACATCTTCACATTCATACTGCTGGGCAAGAAGACGACT
GTTTTTCTCGGTACAAAGGGAAATGATTTCATTTTGAACGGCAAGCTCAAGGATGTTTGCGCGG
AGGATGTCTACTCCCCCCTCACCACCCCAGTGTTCGGACGACATGTGGTGTATGATTGCCCAAA
CTCCAAGCTCATGGAGCAGAAGAAGGTGTGTACAGCAATTTATTCCCGTCTACATCTCCTGGGC
CTCCATTGCTAATTTAATCGTCTACCAGTTCGTCAAGTTCGGCCTCACCTCTGAAGCTCTCCGA
TCCTATGTCACCCTGATCACCAAGGAAGTTGAGCAGTTCTTCGAGTCCTCCCCCGTCTTCAAGG
GCGACTCCGGAGTTTTCAACGTCAGCAAGGTCATGGCTGAAATCACCATCTACACCGCCTCTCG
ATCTCTACAGGGCAAGGAGGTGCGAGGAAAGTTCGATTCCAGCTTTGCGGAACTCTACTCCGAT
CTCGACATGGGCTTCGCCGCCATCAACTTCATGTTCCCATGGTTCCCCTTCCCACACAACCGCA
AGCGTGACCGTGCTCAAAAGAAGATGGCCCAGGTTTACACCGACATCATCCGTCAGCGACGTGA
GGCTGGTGGAGAGAAAGACTCCGAGGACATGGTATGGAACTTGATGTCGTCCGTGTACAAGAAT
GGAACGCCAATTCCAGATATCGAAGTCGCCCACATGATGATTGCTCTTCTTATGGCTGGCCAGC
ACTCTTCTTCCTCCACCGGCTCCTGGATCGTTCTCCGCCTTGCCAGCCGTCCAGATATTCTCGA
GGAACTCTACGAGGAACAGAAACGTGTTCTCGGCGAGGATCTTCCACCACTCACCTACGAATCT
CTCCAGAAACTTGATCTTCACAACAATGTAATCAAGGAGACTCTCCGCCTCCACGCTCCCATCC
ACTCTATCCTCCGTGCTGTTAAATCCCCTATGCCCGTTGAAGGAACTAACTATGTTGTCCCAAC
CTCTCACAACCTCCTTGCCGCTCCTGGTGTTCCCTCACGAGACCCTCAGTACTTCCCTGACCCT
CTTGTTTGGAACCCTCACCGATGGGAGAACAACGTCGGTGTCACCGTAGTCGAGGCCAGTGAAG
AAAAGACAGATTACGAATACGGTTTGGTTAGCAAGGGTGCCAACAGCCCTTACCTCCCATTCGG
CTCAGGCAGACACAGATGCATCGGTGAACAATTTGCATATGTTCAGCTTGGAACCGTAACAGCT
ACGTTAGCCAGACTAATGAAATGGAAGCAAGTTGAAGGCACCAAAGATGTTGTGCCGCCAACTG
ACTATTCGGTAAGTTTGGCATATTACGCTCCTTTTTATTTTGCTCGATTTTACTAACTTTGCAT
TAATAGTCCCTCTTCTCGAAGCCCCTTGGCGACCCAATGGTCTCGTGGGAGAAGCGAAAGCAGC
AGAAAAACCGGCTTGGTCGCTGCCTTGGAGACGACGGCGATTCTCGTGTT**TAG**GATTTCATTTT
TGAGTGATATAACTATGGGTACAATAGCGTCCACTGGGCAATGTAATTACAGTCTCTTCTCACT
CACCACCGGACAATAATAACAATATTAATATTGACGGACCGTTGATAGCTTAACCTGCCATGAC
TAAGCCGGCCTCCCTGAAGCCGCGATGGCCTGATGATGCGCCCACCGTCAGGTCTCGCTCGCTC
AGGCAGCAGAGAATCCAGCGGAGGAGATGACGCCCTGCACGAACACCGTCCAGGGACCGTCTCC

GCCCCGTCGCGAGCTCGAATTGTAATCATCTCGACGGCCGTTGGCCTGCTCGCCGCAAGGGAGA
AAAAGGGGGGCAGAAGGGCAGCGGTAATTTTGGTATTGGAGATGCAGCTGTAAACCAATAAAAA
ACGACGTAAAATACCAGGAACAATGCATTATCTTACAGTACCGTACGCCCCTTGCATAAAATGC
GAGTTCGATCTCAAGATGCAACTTTTTTTTTCCCTTGCTTCGTCCGCAGCAGCTTCAAGGCATT
CATCACAAACAACGAACAAGTCTTGAGCGGAAGCAGGAGGATAAGACGATCACC**ATG**GGCCTCT
TAGCCGACATTGTCTCTCGTTTCTGCGAGAACTGCTCGACCCTGTCCACCGCCGCGCTCGTCGC
AAGTGCCGTATCGGCTTTTATCGTCCTCTCCATTGTTATCAACGTCCTGCAGCAGCTCCTGTTC
AAGGACCCTACAAAGCCTCCGGTGGTCTTCCACTGGGTTCCGGTCATTGGAAGCACGATCTCCT
ATGGAATTGACCCGTACAAGTTCTTTGACGACTGCAAGGAGAAGGTTAGTTGGTGCAATTCAAC
TTAGCTATATACCAGGTTGCGCAAAGCTAACGTATAAATAATTTCGGTACTCTAGTATGGAGAC
ATCTTCACATTCATACTGCTGGGCAAGAAGACGACTGTTTTTCTCGGTACAAAGGGAAATGATT
TCATTTTGAACGGCAAGCTCAAGGATGTTTGCGCGGAGGATGTCTACTCCCCCCTCACCACCCC
AGTGTTCGGACGACATGTGGTGTATGATTGCCCAAACTCCAAGCTCATGGAGCAGAAGAAGGTG
TGTACAGCAATTTATTCCCGTCTACATCTCCTGGGCCTCCATTGCTAATTTAATCGTCTACCAG
TTCGTCAAGTTCGGCCTCACCTCTGAAGCTCTCCGATCCTATGTCACCCTGATCACCAAGGAAG
TTGAGCAGTTCTTCGAGTCCTCCCCCGTCTTCAAGGGCGACTCCGGAGTTTTCAACGTCAGCAA
GGTCATGGCTGAAATCACCATCTACACCGCCTCTCGATCTCTACAGGGCAAGGAGGTGCGAGGA
AAGTTCGATTCCAGCTTTGCGGAACTCTACTCCGATCTCGACATGGGCTTCGCCGCCATCAACT
TCATGTTCCCATGGTTCCCCTTCCCACACAACCGCAAGCGTGACCGTGCTCAAAAGAAGATGGC
CCAGGTTTACACCGACATCATCCGTCAGCGACGTGAGGCTGGTGGAGAGAAAGACTCCGAGGAC
ATGGTATGGAACTTGATGTCGTCCGTGTACAAGAATGGAACGCCAATTCCAGATATCGAAGTCG
CCCACATGATGATTGCTCTTCTTATGGCTGGCCAGCACTCTTCTTCCTCCACCGGCTCCTGGAT
CGTTCTCCGCCTTGCCAGCCGTCCAGATATTCTCGAGGAACTCTACGAGGAACAGAAACGTGTT
CTCGGCGAGGATCTTCCACCACTCACCTACGAATCTCTCCAGAAACTTGATCTTCACAACAATG
TAATCAAGGAGACTCTCCGCCTCCACGCTCCCATCCACTCTATCCTCCGTGCTGTTAAATCCCC
TATGCCCGTTGAAGGAACTAACTATGTTGTCCCAACCTCTCACAACCTCCTTGCCGCTCCTGGT
GTTCCCTCACGAGACCCTCAGTACTTCCCTGACCCTCTTGTTTGGAACCCTCACCGATGGGAGA
ACAACGTCGGTGTCACCGTAGTCGAGGCCAGTGAAGAAAAGACAGATTACGAATACGGTTTGGT
TAGCAAGGGTGCCAACAGCCCTTACCTCCCATTCGGCTCAGGCAGACACAGATGCATCGGTGAA
CAATTTGCATATGTTCAGCTTGGAACCGTAACAGCTACGTTAGCCAGACTAATGAAATGGAAGC
AAGTTGAAGGCACCAAAGATGTTGTGCCGCCAACTGACTATTCGGTAAGTTTGGCATATTACGC
TCCTTTTTATTTTGCTCGATTTTACTAACTTTGCATTAATAGTCCCTCTTCTCGAAGCCCCTTG
GCGACCCAATGGTCTCGTGGGAGAAGCGAAAGCAGCAGAAAAACCGGCTTGGTCGCTGCCTTGG
AGACGACGGCGATTCTCGTGTT**TAG**GATTTCATTTTTGAGTGATATAACTATGGGTACAATAGC
GTCCACTGGGCAATGTAATTACAGTCTCTTCTCACTCACCACCGGACAATAATAACAATATTAA
TATTGACGGACCGTTGATAGCTTAACCTGCCATGACTAAGCCGGCCTCCCTGAAGCCGCGATGG
CCTGATGATGCGCCCACCGTCAGGTCTCGCTCGCTCAGGCAGCAGAGAATCCAGCGGAGGAGAT
GACGCCCTGCACGAACACCGTCCAGGGACCGTCTCCGCCCCGTCGCGAGCTCGAATTGTAATCA
TCTCGACGGCCGTTGGCCTGCTCGCCGCAAGGGAGAAAAAGGGGGGCAGAAGGGCAGCGGTAAT
TTTGGTATTGGAGATGCAGCTGTAAACCAATAAAAAACGACGTAAAATACCAGGAACAATGCAT
TATCTTACAGTACCGTACGCCCCTTGCATAAAATGCGAGTTCGATCTCAAGATGCAACTTTTTT
TTTCCCTTGCTTCGTCCGCAGCAGCTTCAAGGCATTCATCACAAACAACGAACAAGTCTTGAGC
GGAAGCAGGAGGATAAGACGATCACCATGGGCCTCTTAGCCGACATTGTCTCTCGTTTCTGCGA
GAACTGCTCGACCCTGTCCACCGCCGCGCTCGTCGCAAGTGCCGTATCGGCTTTTATCGTCCTC
TCCATTGTTATCAACGTCCTGCAGCAGCTCCTGTTCAAGGACCCTACAAAGCCTCCGGTGGTCT
TCCACTGGGTTCCGGTCATTGGAAGCACGATCTCCTATGGAATTGACCCGTACAAGTTCTTTGA
CGACTGCAAGGAGAAGGTTAGTTGGTGCAATTCAACTTAGCTATATACCAGGTTGCGCAAAGCT
AACGTATAAATAATTTCGGTACTCTAGTATGGAGACATCTTCACATTCATACTGCTGGGCAAGA
AGACGACTGTTTTTCTCGGTACAAAGGGAAATGATTTCATTTTGAACGGCAAGCTCAAGGATGT
TTGCGCGGAGGATGTCTACTCCCCCCTCACCACCCCAGTGTTCGGACGACATGTGGTGTATGAT
TGCCCAAACTCCAAGCTCATGGAGCAGAAGAAGGTGTGTACAGCAATTTATTCCCGTCTACATC
TCCTGGGCCTCCATTGCTAATTTAATCGTCTACCAGTTCGTCAAGTTCGGCCTCACCTCTGAAG

CTCTCCGATCCTATGTCACCCTGATCACCAAGGAAGTTGAGCAGTTCTTCGAGTCCTCCCCCGT
CTTCAAGGGCGACTCCGGAGTTTTCAACGTCAGCAAGGTCATGGCTGAAATCACCATCTACACC
GCCTCTCGATCTCTACAGGGCAAGGAGGTGCGAGGAAAGTTCGATTCCAGCTTTGCGGAACTCT
ACTCCGATCTCGACATGGGCTTCGCCGCCATCAACTTCATGTTCCCATGGTTCCCCTTCCCACA
CAACCGCAAGCGTGACCGTGCTCAAAAGAAGATGGCCCAGGTTTACACCGACATCATCCGTCAG
CGACGTGAGGCTGGTGGAGAGAAAGACTCCGAGGACATGGTATGGAACTTGATGTCGTCCGTGT
ACAAGAATGGAACGCCAATTCCAGATATCGAAGTCGCCCACATGATGATTGCTCTTCTTATGGC
TGGCCAGCACTCTTCTTCCTCCACCGGCTCCTGGATCGTTCTCCGCCTTGCCAGCCGTCCAGAT
ATTCTCGAGGAACTCTACGAGGAACAGAAACGTGTTCTCGGCGAGGATCTTCCACCACTCACCT
ACGAATCTCTCCAGAAACTTGATCTTCACAACAATGTAATCAAGGAGACTCTCCGCCTCCACGC
TCCCATCCACTCTATCCTCCGTGCTGTTAAATCCCCTATGCCCGTTGAAGGAACTAACTATGTT
GTCCCAACCTCTCACAACCTCCTTGCCGCTCCTGGTGTTCCCTCACGAGACCCTCAGTACTTCC
CTGACCCTCTTGTTTGGAACCCTCACCGATGGGAGAACAACGTCGGTGTCACCGTAGTCGAGGC
CAGTGAAGAAAAGACAGATTACGAATACGGTTTGGTTAGCAAGGGTGCCAACAGCCCTTACCTC
CCATTCGGCTCAGGCAGACACAGATGCATCGGTGAACAATTTGCATATGTTCAGCTTGGAACCG
TAACAGCTACGTTAGCCAGACTAATGAAATGGAAGCAAGTTGAAGGCACCAAAGATGTTGTGCC
GCCAACTGACTATTCGGTAAGTTTGGCATATTACGCTCCTTTTTATTTTGCTCGATTTTACTAA
CTTTGCATTAATAGTCCCTCTTCTCGAAGCCCCTTGGCGACCCAATGGTCTCGTGGGAGAAGCG
AAAGCAGCAGAAAAACCGGCTTGGTCGCTGCCTTGGAGACGACGGCGATTCTCGTGTT**TAG**GAT
TTCATTTTTGAGTGATATAACTATGGGTACAATAGCGTCCACTGGGCAATGTAATTACAGTCTC
TTCTCACTCACCACCGGACAATAATAACAATATTAATATTGACGGACCGTTGATAGCTTAACCT
GCCATGACTAAGCCGGCCTCCCTGAAGCCGCGATGGCCTGATGATGCGCCCACCGTCAGGTCTC
GCTCGCTCAGGCAGCAGAGAATCCAGCGGAGGAGATGACGCCCTGCACGAACACCGTCCAGGGA
CCGTCTCCGCCCCGTCGCGAGCTCGAATTGTAATCATCTCGACGGCCGTTGGCCTGCTCGCCGC
AAGGGAGAAAAAGGGGGGCAGAAGGGCAGCGGTAATTTTGGTATTGGAGATGCAGCTGTAAACC
AATAAAAAACGACGTAAAATACCAGGAACAATGCATTATCTTACAGTACCGTACGCCCCTTGCA
TAAAATGCGAGTTCGATCTCAAGATGCAACTTTTTTTTTCCCTTGCTTCGTCCGCAGCAGCTTC
AAGGCATTCATCACAAACAACGAACAAGTCTTGAGCGGAAGCAGGAGGATAAGACGATCACC**AT**
GGGCCTCTTAGCCGACATTGTCTCTCGTTTCTGCGAGAACTGCTCGACCCTGTCCACCGCCGCG
CTCGTCGCAAGTGCCGTATCGGCTTTTATCGTCCTCTCCATTGTTATCAACGTCCTGCAGCAGC
TCCTGTTCAAGGACCCTACAAAGCCTCCGGTGGTCTTCCACTGGGTTCCGGTCATTGGAAGCAC
GATCTCCTATGGAATTGACCCGTACAAGTTCTTTGACGACTGCAAGGAGAAGGTTAGTTGGTGC
AATTCAACTTAGCTATATACCAGGTTGCGCAAAGCTAACGTATAAATAATTTCGGTACTCTAGT
ATGGAGACATCTTCACATTCATACTGCTGGGCAAGAAGACGACTGTTTTTCTCGGTACAAAGGG
AAATGATTTCATTTTGAACGGCAAGCTCAAGGATGTTTGCGCGGAGGATGTCTACTCCCCCCTC
ACCACCCCAGTGTTCGGACGACATGTGGTGTATGATTGCCCAAACTCCAAGCTCATGGAGCAGA
AGAAGGTGTGTACAGCAATTTATTCCCGTCTACATCTCCTGGGCCTCCATTGCTAATTTAATCG
TCTACCAGTTCGTCAAGTTCGGCCTCACCTCTGAAGCTCTCCGATCCTATGTCACCCTGATCAC
CAAGGAAGTTGAGCAGTTCTTCGAGTCCTCCCCCGTCTTCAAGGGCGACTCCGGAGTTTTCAAC
GTCAGCAAGGTCATGGCTGAAATCACCATCTACACCGCCTCTCGATCTCTACAGGGCAAGGAGG
TGCGAGGAAAGTTCGATTCCAGCTTTGCGGAACTCTACTCCGATCTCGACATGGGCTTCGCCGC
CATCAACTTCATGTTCCCATGGTTCCCCTTCCCACACAACCGCAAGCGTGACCGTGCTCAAAAG
AAGATGGCCCAGGTTTACACCGACATCATCCGTCAGCGACGTGAGGCTGGTGGAGAGAAAGACT
CCGAGGACATGGTATGGAACTTGATGTCGTCCGTGTACAAGAATGGAACGCCAATTCCAGATAT
CGAAGTCGCCCACATGATGATTGCTCTTCTTATGGCTGGCCAGCACTCTTCTTCCTCCACCGGC
TCCTGGATCGTTCTCCGCCTTGCCAGCCGTCCAGATATTCTCGAGGAACTCTACGAGGAACAGA
AACGTGTTCTCGGCGAGGATCTTCCACCACTCACCTACGAATCTCTCCAGAAACTTGATCTTCA
CAACAATGTAATCAAGGAGACTCTCCGCCTCCACGCTCCCATCCACTCTATCCTCCGTGCTGTT
AAATCCCCTATGCCCGTTGAAGGAACTAACTATGTTGTCCCAACCTCTCACAACCTCCTTGCCG
CTCCTGGTGTTCCCTCACGAGACCCTCAGTACTTCCCTGACCCTCTTGTTTGGAACCCTCACCG
ATGGGAGAACAACGTCGGTGTCACCGTAGTCGAGGCCAGTGAAGAAAAGACAGATTACGAATAC
GGTTTGGTTAGCAAGGGTGCCAACAGCCCTTACCTCCCATTCGGCTCAGGCAGACACAGATGCA

TCGGTGAACAATTTGCATATGTTCAGCTTGGAACCGTAACAGCTACGTTAGCCAGACTAATGAA
ATGGAAGCAAGTTGAAGGCACCAAAGATGTTGTGCCGCCAACTGACTATTCGGTAAGTTTGGCA
TATTACGCTCCTTTTTATTTTGCTCGATTTTACTAACTTTGCATTAATAGTCCCTCTTCTCGAA
GCCCCTTGGCGACCCAATGGTCTCGTGGGAGAAGCGAAAGCAGCCTTCCCAGAAA**TGA**TAATCA
CGACTGCGGATTGAATCATGTTAAATTTCTTGCTTCTTGCTAATTTATTTCTTCTCTTTTCTTA
CGTTTATACACCGAGCCATATTTGCACTTTATGGTCACGCAAATAGGTTTTCGGAATAGAGCAT
ATGTAATAAAAACTTTCTTTTGACCATGCTTTTGAAGATGGGGGATGTAGATAATAGTATTATT
CTAATACAGCCATTTATTACAATACTCTAGATGAACAGGAAAGTTGCAGTTACTCCGTTAGCTT
TGGTGCAGAAACAAAATGGTAATATACATTGGAAATAGTACATCTTCCCGTAGCTGGGATAATC
TAGCATCTAAACAGCGATATCCAAGCCAGTAATGTCAGGAAGTAACCAGCCAGCAACGTCGTTA
ATCCCGTGCCCCCTTTCAACTCCGACGCTACAAAACGCCTAAGCAGCTGCCTGAGCATTCCTCC
TCTCCGCCAACGCTCTCTTCGCTCTGATCTCCTTCTGTCTTCTCTGACCACCGTCAATCTTTCT
GCCACGCTTATGCTCCCACATGCCATCAGTAACCTGAACCTTGACGGCGGTACTCCATGTGTTT
GGAATAATCTGAGCATACGGCCCCTTGATCATATTCCCGTGGTGCAATTTATAATGGGCAAACT
TTTTGAGTGGCTTGGAAGTATCCTTGAGCTCTGTTTCGCCTGGTGGTAGATTGACAATCGCATA
ACGCATTCCAGGTGTAAGGGTAGCGGTGCCCATTGTGCTAACATCTAGGGACTCTCCGTCGGTG
GCATTGGTAGTTTTTGCACTCTTTGACTTTTCGACGGGGACCTCAACAGCTCTCAGCTCAGCAA
CACCGTCAACAACGTTTTCGAAATCACCTCCTGGGCCGTAGTCCTGCTGTCTGAATTTCTCTCT
CCACCGCAGTATGTATCGTCTGTCCCTTGTCATTTCTATGCCCAATTCTCGAAGCTCAGCCGAG
TCCGCTGTAAACAATTGCTTCCATCCGGTGATTTTGCTGGAATATCTGGACATGTTTCGCCCAA
TTAGGCGTAGGAATGTTTCGACATCGGGCACGAATGGGGTAGGTGATGGCACTGTTGCGGGTTT
TCGAGATTGATGAAGGTACCGTACGCATGGCTGATAATGGGGGTTTGTGACGGAAGTGAAGAGC
CGTAGTGGAAGGGATGTTCTGGCATTAAAGCCCGGTATGCGCGCCGCAGCCATAGTGGTTATAT
CGCCTTATATCAAACCGTTCAATGTCGAGATCTGGGGGGTGAAGATTTTAATTTAGCTCGTGGT
GTAAGAACACCATCGGCGGGCGTGACGAAAGCGAAAAAAGCCACGGCACCAAGGGCCCAGGTAA
ACCGGTAAGGGACTCAAGGCGAGTCAATCCAATGCTCAATGGCGCTGTATAGAAGATGTTCAAC
TGGTTCTTCGACTTCGTTCAAGATTTTGCACCAAAATTTTGAATTAGGTCCGACGGCCTTAGCT
CGGATCAACAAAACGCCGATCTCCGGTGAGCTCGGGCCAGCCGAAGCTTCCATCGCAACATTCA
AAGATGAAAGACGACGATATATATTTTGATAACAAACAAAGCTGTATTGTTGATTGAATACTAG
GAGAGCTGCTCAGAGCAGTGTCTCACTCTTGTGCCACTCTATCCTTTGTCTCGCGAAATTGGTG
GAAGCCAATTTCGCTTGACATTATGGCAGTGTCGCCGCTTCCCTCTCAATTCCCTCCCATCACT
ACTACCGGTGCTCCAAAGTCGTTAGCGATGAGAAATATTGCTTCTCAAGAGCCTCGGACGGCGC
CTAGTGGTGATCCTGCAGTTCCTAAGCCCTCCATGCCCGAACCAGAGCTGGCGGAGCAGTTAAA
CGCTGATGTACGGCGGAAATATGTCAAAGGTGGGCGGGCAAATACACCCCAACCATTGGGACCC
CATAATGCTAACATTTGGTTATTCTGAAAGATAAGAAGCTCGGTGAAGGTACATATGCAGTTGT
TTACCTAGGCCATCTACGAGATGATCCCACTTCGCTCGTTGCAATCAAGAAAATAAAACTAAAC
GCCGAGTACAAGGACGGGCTATCCATGGACGCCATCCGTGAAGTAAAGTACCTTCAGGAACTGT
CTCACCCGAACGTAATTGCGTTGCATGACGTTTTCTCCTCAAAGGATCAAAATCTAAACCTCGT
CCTTGAATTTTTACCCCTTGGAGATCTTGAAATGTTGATCAAAGACAACTCTATCCAGTACGGT
GTGGCTGATATAAAAGCATGGATAAGCATGCTTGCACGTGGAGTGTGGTTCTGTCATAAGAATT
TCATTCTACACCGTGATATTAAGCCTAACAACTTATTAATTGCATCGGATGGAGAAGTTAAATT
AGCGGATTTTGGTCTTGCCAGATCCTTTGCCGATCCATATCTCAACATGACACATCAAGTCATC
ACAAGATGGTACAGGCCATTAGAACTACTCTTCGGCGCCAGGCAATATTCCGGTGCAGTCGACA
TATGGTCTATGGG

Protein alignments:
Green: Typical cytochrome P450 monooxygenases ER anchoring TM
Blue: Inhibitor binding sites (from A. fumigatus)
Red: Heme-binding cysteine
Gray: New C-terminus of the additional copies.

```
MGLLADIVSRFCENCSTLSTAALVASAVSAFIVLSIVINVLQQLLFKDPTKPPVVFHWVPVIGS
MGLLADIVSRFCENCSTLSTAALVASAVSAFIVLSIVINVLQQLLFKDPTKPPVVFHWVPVIGS
MGLLADIVSRFCENCSTLSTAALVASAVSAFIVLSIVINVLQQLLFKDPTKPPVVFHWVPVIGS
MGLLADIVSRFCENCSTLSTAALVASAVSAFIVLSIVINVLQQLLFKDPTKPPVVFHWVPVIGS
MGLLADIVSRFCENCSTLSTAALVASAVSAFIVLSIVINVLQQLLFKDPTKPPVVFHWVPVIGS
MGLLADIVSRFCENCSTLSTAALVASAVSAFIVLSIVINVLQQLLFKDPTKPPVVFHWVPVIGS
MGLLADIVSRFCENCSTLSTAALVASAVSAFIVLSIVINVLQQLLFKDPTKPPVVFHWVPVIGS

TISYGIDPYKFFDDCKEKYGDIFTFILLGKKTTVFLGTKGNDFILNGKLKDVCAEDVYSPLTTP
TISYGIDPYKFFDDCKEKYGDIFTFILLGKKTTVFLGTKGNDFILNGKLKDVCAEDVYSPLTTP
TISYGIDPYKFFDDCKEKYGDIFTFILLGKKTTVFLGTKGNDFILNGKLKDVCAEDVYSPLTTP
TISYGIDPYKFFDDCKEKYGDIFTFILLGKKTTVFLGTKGNDFILNGKLKDVCAEDVYSPLTTP
TISYGIDPYKFFDDCKEKYGDIFTFILLGKKTTVFLGTKGNDFILNGKLKDVCAEDVYSPLTTP
TISYGIDPYKFFDDCKEKYGDIFTFILLGKKTTVFLGTKGNDFILNGKLKDVCAEDVYSPLTTP
TISYGIDPYKFFDDCKEKYGDIFTFILLGKKTTVFLGTKGNDFILNGKLKDVCAEDVYSPLTTP

VFGRHVVYDCPNSKLMEQKKFVKFGLTSEALRSYVTLITKEVEQFFESSPVFKGDSGVFNVSKV
VFGRHVVYDCPNSKLMEQKKFVKFGLTSEALRSYVTLITKEVEQFFESSPVFKGDSGVFNVSKV
VFGRHVVYDCPNSKLMEQKKFVKFGLTSEALRSYVTLITKEVEQFFESSPVFKGDSGVFNVSKV
VFGRHVVYDCPNSKLMEQKKFVKFGLTSEALRSYVTLITKEVEQFFESSPVFKGDSGVFNVSKV
VFGRHVVYDCPNSKLMEQKKFVKFGLTSEALRSYVTLITKEVEQFFESSPVFKGDSGVFNVSKV
VFGRHVVYDCPNSKLMEQKKFVKFGLTSEALRSYVTLITKEVEQFFESSPVFKGDSGVFNVSKV
VFGRHVVYDCPNSKLMEQKKFVKFGLTSEALRSYVTLITKEVEQFFESSPVFKGDSGVFNVSKV

MAEITIYTASRSLQGKEVRGKFDSSFAELYSDLDMGFAAINFMFPWFPFPHNRKRDRAQKKMAQ
MAEITIYTASRSLQGKEVRGKFDSSFAELYSDLDMGFAAINFMFPWFPFPHNRKRDRAQKKMAQ
MAEITIYTASRSLQGKEVRGKFDSSFAELYSDLDMGFAAINFMFPWFPFPHNRKRDRAQKKMAQ
MAEITIYTASRSLQGKEVRGKFDSSFAELYSDLDMGFAAINFMFPWFPFPHNRKRDRAQKKMAQ
MAEITIYTASRSLQGKEVRGKFDSSFAELYSDLDMGFAAINFMFPWFPFPHNRKRDRAQKKMAQ
MAEITIYTASRSLQGKEVRGKFDSSFAELYSDLDMGFAAINFMFPWFPFPHNRKRDRAQKKMAQ
MAEITIYTASRSLQGKEVRGKFDSSFAELYSDLDMGFAAINFMFPWFPFPHNRKRDRAQKKMAQ

VYTDIIRQRREAGGEKDSEDMVWNLMSSVYKNGTPIPDIEVAHMMIALLMAGQHSSSSTGSWIV
VYTDIIRQRREAGGEKDSEDMVWNLMSSVYKNGTPIPDIEVAHMMIALLMAGQHSSSSTGSWIV
VYTDIIRQRREAGGEKDSEDMVWNLMSSVYKNGTPIPDIEVAHMMIALLMAGQHSSSSTGSWIV
VYTDIIRQRREAGGEKDSEDMVWNLMSSVYKNGTPIPDIEVAHMMIALLMAGQHSSSSTGSWIV
VYTDIIRQRREAGGEKDSEDMVWNLMSSVYKNGTPIPDIEVAHMMIALLMAGQHSSSSTGSWIV
VYTDIIRQRREAGGEKDSEDMVWNLMSSVYKNGTPIPDIEVAHMMIALLMAGQHSSSSTGSWIV
VYTDIIRQRREAGGEKDSEDMVWNLMSSVYKNGTPIPDIEVAHMMIALLMAGQHSSSSTGSWIV

LRLASRPDILEELYEEQKRVLGEDLPPLTYESLQKLDLHNNVIKETLRLHAPIHSILRAVKSPM
LRLASRPDILEELYEEQKRVLGEDLPPLTYESLQKLDLHNNVIKETLRLHAPIHSILRAVKSPM
```

LRLASRPDILEELYEEQKRVLGEDLPPLTYESLQKLDLHNNVIKETLRLHAPIHSILRAVKSPM
LRLASRPDILEELYEEQKRVLGEDLPPLTYESLQKLDLHNNVIKETLRLHAPIHSILRAVKSPM
LRLASRPDILEELYEEQKRVLGEDLPPLTYESLQKLDLHNNVIKETLRLHAPIHSILRAVKSPM
LRLASRPDILEELYEEQKRVLGEDLPPLTYESLQKLDLHNNVIKETLRLHAPIHSILRAVKSPM
LRLASRPDILEELYEEQKRVLGEDLPPLTYESLQKLDLHNNVIKETLRLHAPIHSILRAVKSPM

PVEGTNYVVPTSHNLLAAPGVPSRDPQYFPDPLVWNPHRWENNVGVTVVEASEEKTDYEYGLVS
PVEGTNYVVPTSHNLLAAPGVPSRDPQYFPDPLVWNPHRWENNVGVTVVEASEEKTDYEYGLVS
PVEGTNYVVPTSHNLLAAPGVPSRDPQYFPDPLVWNPHRWENNVGVTVVEASEEKTDYEYGLVS
PVEGTNYVVPTSHNLLAAPGVPSRDPQYFPDPLVWNPHRWENNVGVTVVEASEEKTDYEYGLVS
PVEGTNYVVPTSHNLLAAPGVPSRDPQYFPDPLVWNPHRWENNVGVTVVEASEEKTDYEYGLVS
PVEGTNYVVPTSHNLLAAPGVPSRDPQYFPDPLVWNPHRWENNVGVTVVEASEEKTDYEYGLVS
PVEGTNYVVPTSHNLLAAPGVPSRDPQYFPDPLVWNPHRWENNVGVTVVEASEEKTDYEYGLVS

KGANSPYLPFGSGRHR**C**IGEQFAYVQLGTVTATLARLMKWKQVEGTKDVVPPTDYSSLFSKPLG
KGANSPYLPFGSGRHR**C**IGEQFAYVQLGTVTATLARLMKWKQVEGTKDVVPPTDYSSLFSKPLG
KGANSPYLPFGSGRHR**C**IGEQFAYVQLGTVTATLARLMKWKQVEGTKDVVPPTDYSSLFSKPLG
KGANSPYLPFGSGRHR**C**IGEQFAYVQLGTVTATLARLMKWKQVEGTKDVVPPTDYSSLFSKPLG
KGANSPYLPFGSGRHR**C**IGEQFAYVQLGTVTATLARLMKWKQVEGTKDVVPPTDYSSLFSKPLG
KGANSPYLPFGSGRHR**C**IGEQFAYVQLGTVTATLARLMKWKQVEGTKDVVPPTDYSSLFSKPLG
KGANSPYLPFGSGRHR**C**IGEQFAYVQLGTVTATLARLMKWKQVEGTKDVVPPTDYSSLFSKPLG

DPMVSWEKRKQQKNRLGRCLGDDGDSRV
DPMVSWEKRKQQKNRLGRCLGDDGDSRV
DPMVSWEKRKQQKNRLGRCLGDDGDSRV
DPMVSWEKRKQQKNRLGRCLGDDGDSRV
DPMVSWEKRKQQKNRLGRCLGDDGDSRV
DPMVSWEKRKQQKNRLGRCLGDDGDSRV
DPMVSWEKRKQPSQK
